# Identification of stage-related and severity-related biomarkers and exploration of immune landscape for Dengue by comprehensive analyses

**DOI:** 10.1186/s12985-022-01853-8

**Published:** 2022-08-02

**Authors:** Nan Xiong, Qiangming Sun

**Affiliations:** 1 Institute of Medical Biology, Chinese Academy of Medical Sciences and Peking Union Medical College, Kunming, 650118 People’s Republic of China; 2 Kunming Medical University, Kunming, 650500 People’s Republic of China; 3 Yunnan Key Laboratory of Vaccine Research and Development on Severe Infectious Diseases, Kunming, 650118 People’s Republic of China

**Keywords:** DH, DHF, *CD38*, *ZNF595*, Autophagy

## Abstract

**Background:**

At present, there are still no specific therapeutic drugs and appropriate vaccines for Dengue. Therefore, it is important to explore distinct clinical diagnostic indicators.

**Methods:**

In this study, we combined differentially expressed genes (DEGs) analysis, weighted co-expression network analysis (WGCNA) and Receiver Operator Characteristic Curve (ROC) to screen a stable and robust biomarker with diagnosis value for Dengue patients. CIBERSORT was used to evaluate immune landscape of Dengue patients. Gene Ontology (GO) enrichment, Kyoto Encyclopedia of Genes and Genomes (KEGG) analysis and Gene set enrichment analysis (GSEA) were applied to explore potential functions of hub genes.

**Results:**

*CD38* and Plasma cells have excellent Area Under the Curve (AUC) in distinguishing clinical stages for Dengue patients, and activated memory CD4+ T cells and Monocytes have good AUC for this function. *ZNF595* has acceptable AUC in discriminating dengue hemorrhagic fever (DHF) from dengue fever (DF) in whole acute stages. Analyzing any serotype, we can obtain consistent results. Negative inhibition of viral replication based on GO, KEGG and GSEA analysis results, up-regulated autophagy genes and the impairing immune system are potential reasons resulting in DHF.

**Conclusions:**

*CD38*, Plasma cells, activated memory CD4+ T cells and Monocytes can be used to distinguish clinical stages for dengue patients, and *ZNF595* can be used to discriminate DHF from DF, regardless of serotypes.

**Graphical abstract:**

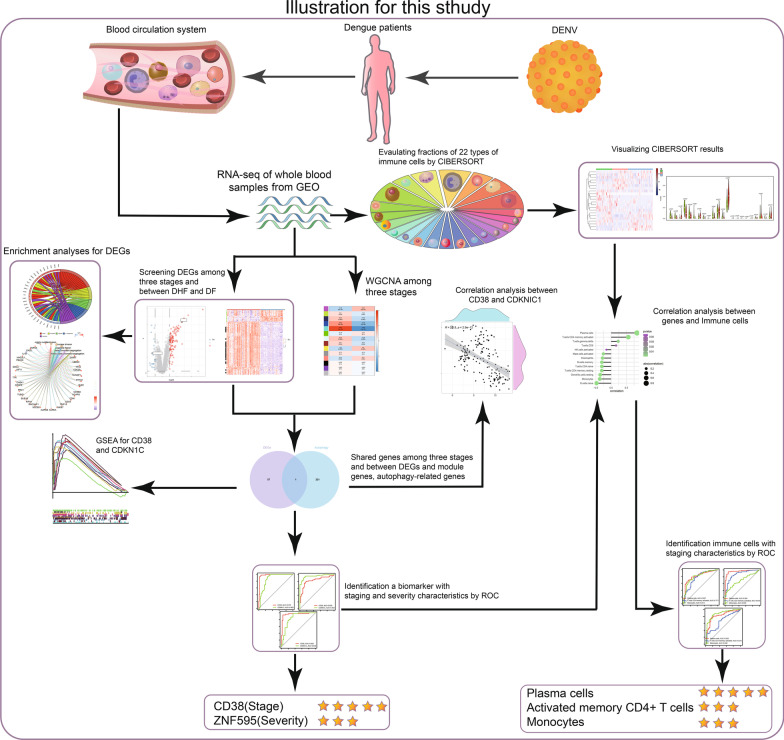

**Supplementary Information:**

The online version contains supplementary material available at 10.1186/s12985-022-01853-8.

## Introduction

Dengue was listed by the World Health Organization (WHO) as one of the top ten global health threats announced at the beginning of 2019 [1]. In the past few decades, Dengue has become the fastest-growing mosquito borne disease in the world [2–4]**,** seriously endangering human health [5, 6]. Vaccine research and development continue to make progress [7–12], but the antibody-dependent enhancement (ADE) limits the effectiveness of vaccines [13–17]. Asymptomatic infections increase incidence of dengue [16, 18] and effective treatments have not been identified. Therefore, it’s urgent to explore the pathogenic mechanism of dengue fever and screen out molecular markers for better diagnosis and treatments.

Autophagy, a catabolic process that degrades damaged or abnormal intracellular components to recover nutrients, is essential for maintaining cell and body homeostasis [19, 20], and benefits to proliferation and infection of the Dengue virus (DENV) [21–24]. In DENV-ADE infection, cross-reactive antibodies mediate infection by inducing autophagy related proteins, and then suppress the innate immunity mediated by the mitochondria antiviral protein (MAVS) [25]. Immune response affects directly or indirectly host response to DENV in varying degrees, including symptomatic infection, asymptomatic infection [26, 27], dengue shock syndrome (DSS) and dengue hemorrhagic fever (DHF) [28–30]. Therefore, it is essential to explore autophagy and immune response during DENV infection.

Transcriptomics researches are beneficial to assist researchers in better understanding disease causes [31] and locating biomarkers [32–34]. Our precious transcriptomics researches contributed to understand viral evolution and its impact on pathogenicity and vaccine development of DENV [35–38]. However, studies [39–41] published focused on multi-gene researches, and single analytical method (differentially expressed genes (DEGs) analyses), and did not link genomics with immune landscapes. In this study, we used a combination of DEG analyses, weighted co-expression network analysis (WGCNA) and Receiver Operator Characteristic Curve (ROC) to identify, validate and test biomarkers with diagnostic value of stages and severity in independent datasets, and applied the CIBERSORT website to analyze immune landscape differences among three stages and between DHF and Dengue Fever (DF) and explore correlations between genes and immune cells.

## Materials and methods

### Data source

Gene expression datasets of Dengue patients were selected from a public database named the Gene Expression Omnibus databases (GEO, http://www.ncbi.nlm.nih.gov/geo). We followed these reference points: 1. datasets analyzed whole blood samples of different stages from Dengue patients including DF and DHF, and normal samples; 2. Datasets should include at least 20 dengue patients. Based on these criteria, we obtained GSE43777 dataset analyzing Two chip types (affymetrix HG-U133 plus 2 in GLP570 platform and HG-Focus in GLP201 platform) [41], GSE28405 dataset [42] and GSE51808 dataset [43].

In GSE43777, more than 200 samples (one sample for each stage) were collected from 51 DF and 13 DHF subjects in Maracay, Venezuela, and demographics and clinical, immunological and hematological characteristics of participants were preciously summarized [41]. In GSE28405, 31 clinically undifferentiated DENV RT-PCR positive patient samples were selected in Singapore within 72 h (Early Acute stage, EA), 4–7 days (Late Acute stage, LA) and 3–4 weeks (Convalescence stage, C) after self-reported fever onset, and other clinical information had been recorded as preciously described [42]. In GSE51808, whole blood samples were obtained from 47 Dengue patients (DF n = 31, DHF = 16) hospitalized at the Siriraj Hospital in Bangkok within days 2 and at 4 weeks or later after discharge (the Convalescence stage) and 9 normal subjects were analyzed， and detail clinical information were also showed [44].

Regarding gene expression levels in C or EA stages as baselines, these patients were divided into 3 groups, C vs EA, C vs LA and EA vs LA, as precious studies [41]. The Principal Component Analysis (PCA) analysis [45] was used to explore whether different stages can be distinguished clearly.

### DEGs analysis and functional annotation of DEGs

A website (http://sangerbox.com/Tool) based on a R package “Limma” was applied to analyze DEGs among three phases (we showed the analysis regardless of serotypes and also performed separate analysis for each serotype) and between DHF and DF for Dengue patients. A R package “clusterProfiler” in R version 4.1.0 and a gene set enrichment analysis (GSEA) software (4.1.0) [46] were used to performed Gene Ontology (GO) enrichment analysis, Kyoto Encyclopedia of Genes and Genomes (KEGG) analysis and GSEA to explore potential bio-function and enrichment pathways for DEGs.

### WGCNA analysis [47]

We obtained a total of 4843 genes in the C vs EA group, 4472 genes in the C vs the LA group and 4463 genes in the EA vs LA group by a “WGCNA” package. We converted the adjacency matrix into the topological overlap matrix (TOM) when the power of β were equal to 5, 4 and 12. According to a height cutoff of 0.25, we merged similar modules and the module displaying the highest conformity with the development disease of Dengue was used to intersect with DEGs to identify more stable DEGs.

### Evaluation immune cells of the whole blood samples

we estimated fractions of immune cells in whole blood samples by the “CIBERSORT” website (https://cibersortx.stanford.edu/index.php) which can be used to input gene expression data and then obtain a fraction estimate of 22 immune cell types in whole blood samples [48].

### ROC analysis

ROC [49] was applied to identify, valid and test value of biomarkers and immune cells in distinguish stages and severity based on Area Under The Curve (AUC).

### Statistical analyses

R software (version 4.1.0) and R. Studio (version 1.9.0) were used to analyze data and visualize results. We applied the t-test, Mann–Whitney U test and the chi-square test to identify differences between two groups. Correlation analyses were showed according to the Pearson and Spearman theories. *P* values < 0.05 was regarded as statistical significance.

## Results

**Fig. 1:**
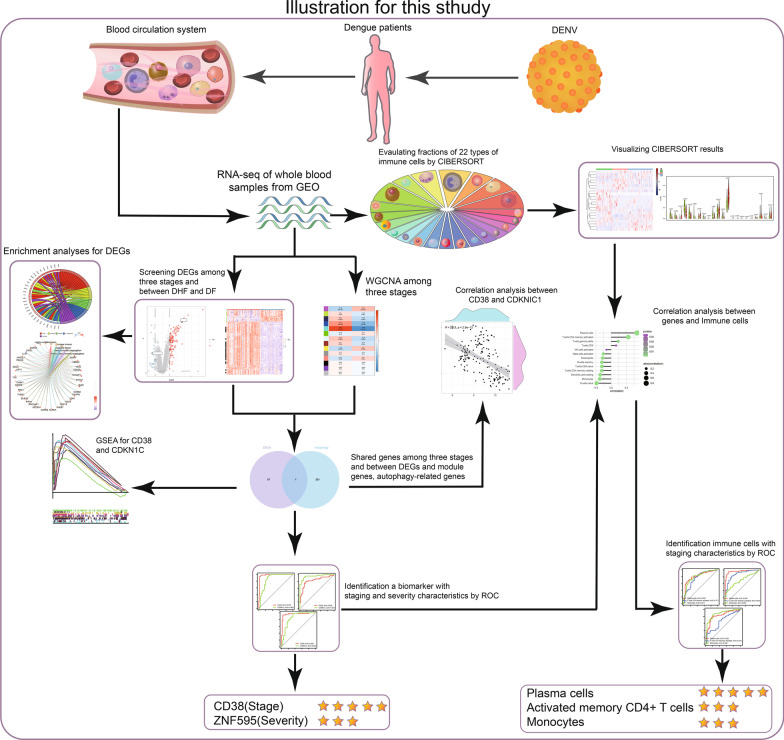
Flow chart diagram. RNA expression levels in peripheral blood mononuclear cell (PBMC) of dengue fever patients will change during Dengue virus (DENV) infection. We downloaded these RNA-sequence data and clinical information from National Center of Biotechnology Information (NCBI) dataset (https://www.ncbi.nlm.nih.gov/geo/). Combining R package “Limma” with “WGCNA”, stable differentially expressed genes (DEGs) among three phases and between DHF and DF for Dengue patients were screened and we also explored potential functions for these DEGs using Gene Ontology (GO), Kyoto Encyclopedia of Genes and Genomes (KEGG) and Gene Set Enrichment Analysis (GSEA) analysis. Based on gene expression data, fractions of immune cells in whole blood samples were estimated by the “CIBERSORT” website and correlations between immune cells and huh genes were also qualified. Finally, we used Area Under the Curve (AUC) to evaluate diagnosis value of huh genes and important immune cells in distinguishing clinical stages and severity

Figure 1 shows illustration for this study.

### Acquisition of DEGs and mechanisms analyses for dengue

**Fig. 2:**
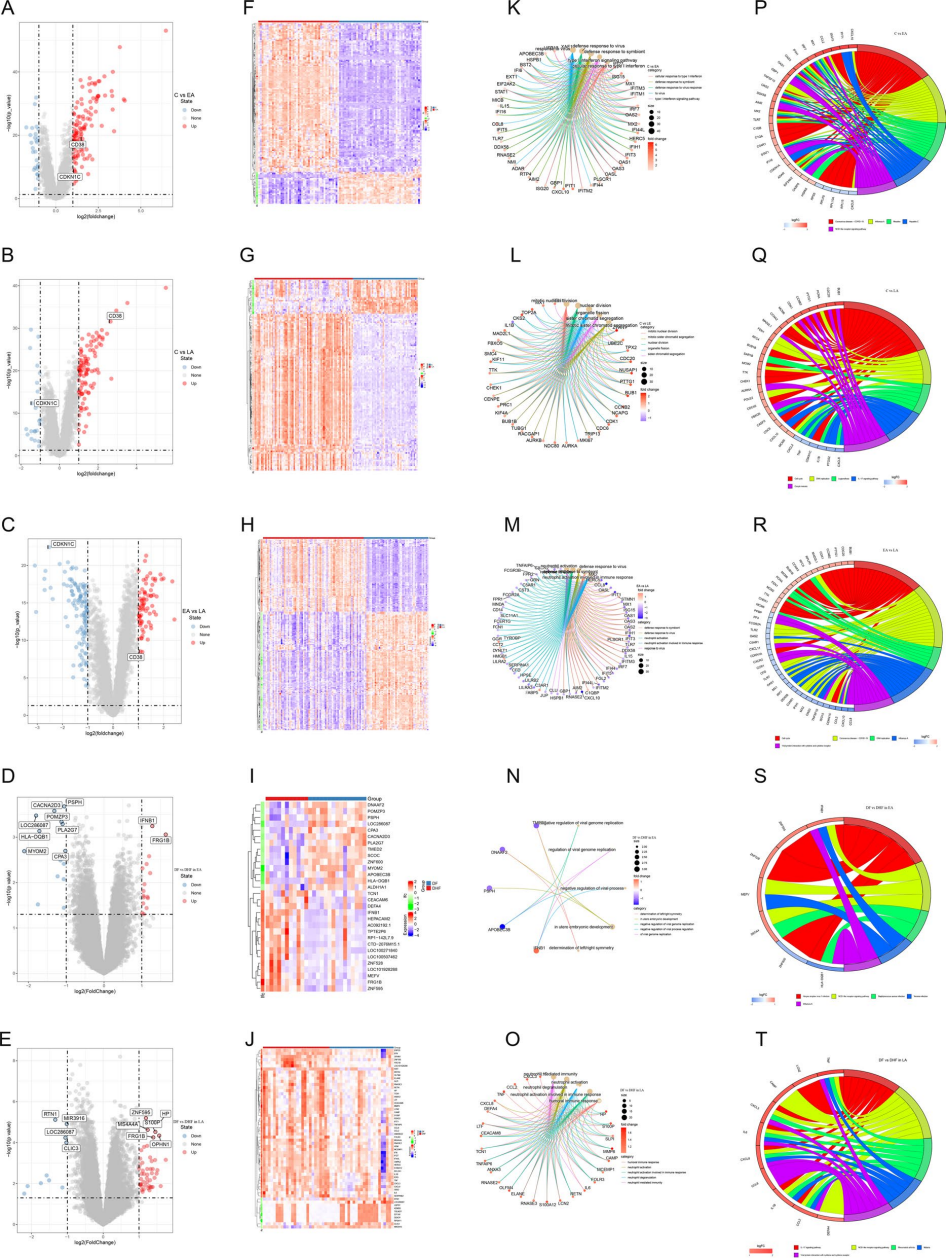
**A–E** Volcano maps and **F–J** heatmaps screen out differentially expressed genes (DEGs). **K–O** Gene Ontology (GO) analysis shows that differentially expressed genes (DEGs) are mainly involved in biological processes and **P–T** Kyoto Encyclopedia of Genes and Genomes (KEGG) analysis displays potential enrichment pathways of DEGs. (C, Convalescent stage; EA, Early Acute stage; LA, Late Acute stage)

The Principal Component Analysis (PCA) shows three stage samples can be distinguished (Additional file 1: Figure S1 A–C). After normalizing the expression data from GSE43777 dataset on GLP201 platform, we identify 147 DEGs (121 up-regulated genes and 26 down-regulated genes) between the C and the EA stages (Fig. 2A, F and Additional file 7: Table S1), 124 DEGs (102 up-regulated genes and 22 down-regulated genes) between the C and the LA stages (Fig. 2B, G and Additional file 8: Table S2) and 195 DEGs (74 up-regulated genes and 121 down-regulated genes) between the EA and LA stages (Fig. 2C, H and Additional file 9: Table S3) by a “Limma” package based on |Log2FC|≥ 1, and adjusted *P* value < 0.05. Likewise, in the EA stage we obtained 30 DEGs (16 up-regulated genes and 14 down-regulated genes) (Fig. 2D, I and Additional file 10: Table S4) between DF (n = 15) and DHF (n = 11) samples from GSE43777 dataset on GLP570 platform, and 58 DEGs (48 up-regulated genes and 10 down-regulated genes) (Fig. 2E, J and Additional file 11: Table S5) between DF (n = 25) and DHF (n = 26) samples in the LA stage, based on |Log2FC|≥ 1 and *P* value < 0.05.

GO enrichment analysis results show that 147 DEGs (between the C and the EA stages) are mainly enriched in defense response to viruses and type I interferon (Fig. 2K); DNA replication is a common bio-activity for 124 DEGs (between the C and the LA stages) (Fig. 2L); the Fig. 2M shows that neutrophil activation and defense response to virus are dominating enrichment biofunctions for 195 DEGs between the EA and LA stages; bio-function involved in negative regulation of viral genome replication are more active for 30 DEGs between DF and DHF samples in Fig. 2N; neutrophil and humoral immune response are activated for DHF samples in the LA stage (Fig. 2O).

GO enrichment analysis results (down-regulated genes in DHF samples enriched in negative regulation of viral replication) in Fig. 2I and N caught our attention, because it suggested it was difficult to control viral replication in DHF patients in the EA stage. Relative studies [21, 24, 50] demonstrated DENV inhibited cell apoptosis through autophagy, thereby promoting its replication. Therefore, we suspecte related-autophagy genes may be activated in EA stage and LA stage. As the Additional file 1: Figure S1 J shown, in the LA stage, we identify definitely 1 different expression autophagy-related gene (*CCL2*) by intersecting DEGs between DHF and DH samples from GSE43777 analyzed on GLP570 platform with 222 autophagy-related genes downloaded from Human Autophagy Database (HADb) which provides a complete and an up-to-date list of human genes and proteins involved directly or indirectly in autophagy. The boxplot diagram (Additional file 1: Figure S1 K) shows that the expression levels of *CCL2* increases significantly in DHF samples in the LA stage, suggesting that DENV replicates more strongly in DHF than DF which may be one reason resulting in DHF in the LA stage*.* While we do not identify different expression of autophagy-related genes between DF and DHF in the EA stage.

KEGG analysis results reveal that pathogen infection and NOD − like receptor signaling pathway are obvious enrichment pathways for 147 DEGs (between the C and the EA stages) (Fig. 2P); Cell replication, Legionellosis and Malaria, are major enrichment pathways for 124 DEGs (between the C and the LA stages) (Fig. 2Q); 195 DEGs (between the EA and LA stages) are enriched in pathogen infection and Cell replication (Fig. 2R); NOD − like receptor signaling pathway and pathogen infection are more active for 30 DEGs between DF (n = 15) and DHF (n = 11) samples in Fig. 2S; inflammatory pathways are activated for DHF samples in LA stage in Fig. 2T.

### Acquisition of key modules by WGCNA

**Fig. 3:**
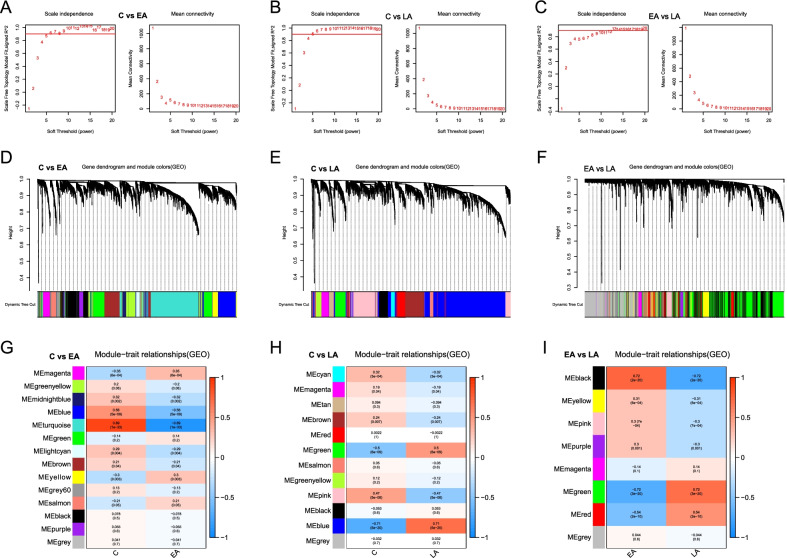
Weighted co-expression network analysis (WGCNA). **A–C** The scale-free fit index (left) and average connectivity (right) of different soft threshold powers. **D–F** Clustering dendrograms of genes. **G–I** Heatmaps of the module-trait display correlations among different stages. (C, Convalescent stage; EA, Early Acute stage; LA, Late Acute stage)

Using the GSE43777 dataset analyzed on GLP201 platform, we performed a “WGCNA” package to obtain key modules associated with procession features of Dengue. We select 5, 4 and 12 (Fig. 3A–C) as the soft-threshold power respectively and 0.25 (Additional file 1: Figure S1 D-F) as the cutoff height in the C vs EA group, in the C vs LA group and in the EA vs LA group. Modules consist of genes with similar expression properties (Additional file 1: Figure S1 G–I). In the cluster tree, we use each branch to represent a gene, and one color to show a co-expression module (Fig. 3D–F).

The association of module eigengene (ME) values and clinical stages is applied to assess the module-stage feature correlation. WGCNA results show 14, 12 and 8 modules, respectively (Fig. 3G–I). Compared the C with the EA group, the turquoise module is the most matched strongly with the EA group (Pearson co-efficient = − 0.89, *P* = 1e−33) and the C group (Pearson co-efficient = 0.89, *P* = 1e−33) in Fig. 3G. Compared the C with the LA group, the most significant correlation can be observed between the LA group (Pearson co-efficient = 0.71, *P* = 5e−20) and the blue module, and between the C group (Pearson co-efficient = − 0.71, P = 5e−20) and the blue module in Fig. 3H. While comparing the LA group with the EA group, the black module and the green module show the same highest correlation (Pearson co-efficient = 0.72) in Fig. 3I. We regard these modules mentioned above as key modules.

### Acquisition of key genes

**Fig. 4:**
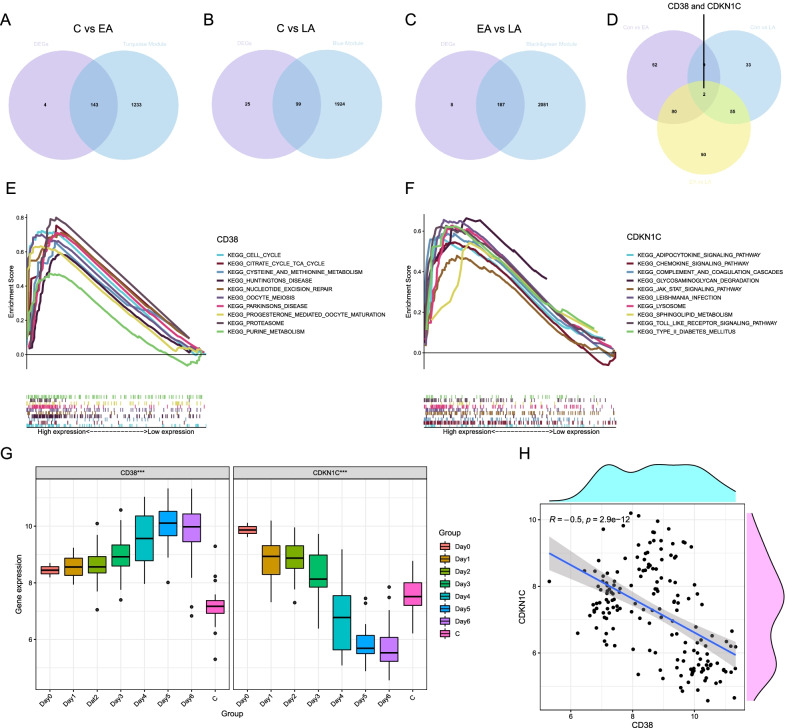
Venn diagrams screen out shared genes: **A** 143 shared genes between the turquoise module and differentially expressed genes (DEGs) (between the C stage and the EA stage); **B** 99 shared genes between the blue module and DEGs (between the C stage and the LA stage); **C** 187 shared genes between the black and green module and DEGs (between the EA stage and the LA stage); **D** 2 shared genes among three stages. **E** and **F** showing the potential function enrichment pathways for *CD38* and *CDKN1C* by gene set enrichment analysis (GSEA). **G** The boxplot showing changes in expression levels of *CD38* and *CDKN1C* over time (***P < 0.001). **H** A negative correlation between *CD38* and *CDKN1C*. (C, Convalescent stage; EA, Early Acute stage; LA, Late Acute stage)

To filter reliable and strong hub genes (shared genes) accurately, Venn diagrams were used to visualize hub genes between DEGs and key modules by a website (http://www.ehbio.com/test/venn/#/) [51]. The results illustrate 143, 99 and 187 hub genes by interacting the turquoise module with 147 DEGs (between the C and the EA stages) (Fig. 4A), interacting the blue module with 124 DEGs (between the C and the LA stages) (Fig. 4B), interacting the black and green modules with 195 DEGs (between the EA and LA stages) (Fig. 4C), respectively. *CD38* and *CDKN1C* are shared by the three phases (Fig. 4D). When considering serotypes, *CD38* and *CDKN1C* still express differentially in three comparison groups (C vs EA, C vs LA, EA vs LA) for each serotype and log|FC|> 1 or log|FC| is extremely closed to 1 (Additional file 12: Table S6). The Fig. 4G shows the expression levels of *CD38* firstly increase and then decrease from day 0 to the C stage (***P < 0.001). While the expression of *CDKN1C* is firstly down-regulated and subsequently up-regulated from the day 0 to the C stage (***P < 0.001) (Fig. 4G). The GSEA shows *CD38* is enriched in cellar proliferation and metabolic pathways (Fig. 4E) and *CDKN1C* is enriched in lipid metabolism and inflammation pathways (Fig. 4F). Correlation analyses show in Fig. 4H expression levels of *CD38* are negatively correlated with *CDKN1C* (spearman correlation = − 0.5). We select *CD38* and *CDKN1C* for follow-up analyses.

### Verification of key genes value in staging diagnosis

**Fig. 5:**
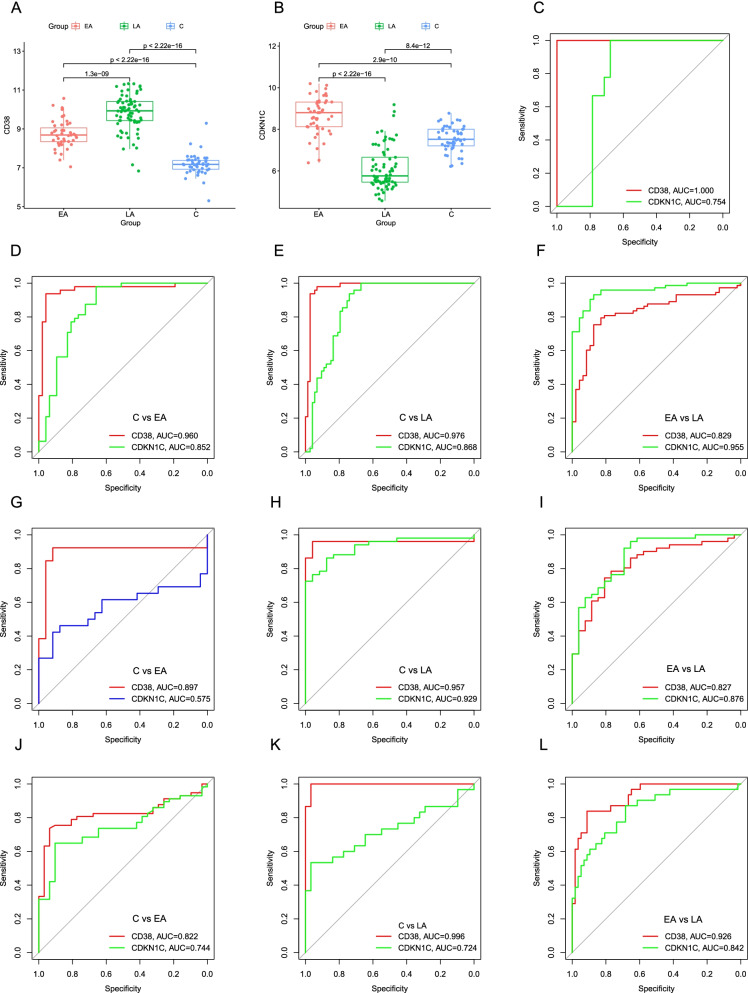
**A** and **B** Significantly different expression levels of *CD38* and *CDKN1C* in three stages. **C** *CD38* and *CDKN1C* can be used to distinguish Dengue samples from normal samples. Analyzing staging diagnosis value of *CD38* and *CDKN1C* for Dengue by Area Under the Curve (AUC): **D–F** analyzed Firstly in the training group, **G–I** verified subsequently in the verifying group, **J–L** tested lastly in the testing group. (C, Convalescent stage; EA, Early Acute stage; LA, Late Acute stage)

Because *CD38* and *CDKN1C* express differently in three stages (Fig. 5A–B), we speculate that they can be used as biomarkers with staging characteristic to distinguish clinical stages for Dengue patients. To identify, verify and test the value of *CD38* and *CDKN1C*, we regard GSE43777 datasets analyzed on the GPL201 platform (C stage, n = 48; EA stage, n = 47; LA stage, n = 73), GSE43777 datasets analyzed on the GPL570 platform (C stage, n = 24; EA stage, n = 26; LA stage, n = 51) and GSE28405 (C stage, n = 31; EA stage, n = 57; LA stage, n = 31) as a training dataset, a verifying dataset and a testing dataset, respectively. When analyzing single serotype, we regard GSE43777 datasets analyzed on the GPL201 platform (Serotype I: C stage, n = 26; EA stage, n = 26; LA stage, n = 44. Serotype II: C stage, n = 6; EA stage, n = 7; LA stage, n = 5. serotype III: C stage, n = 9; EA stage, n = 9; LA stage, n = 15. Serotype IV: C stage, n = 6; EA stage, n = 4; LA stage, n = 8.), GSE43777 datasets analyzed on the GPL570 platform (Serotype I: C stage, n = 7; EA stage, n = 3; LA stage, n = 14. Serotype II: C stage, n = 11; EA stage, n = 16; LA stage, n = 23. serotype III: C stage, n = 3; EA stage, n = 3; LA stage, n = 8. Serotype IV: C stage, n = 3; EA stage, n = 3; LA stage, n = 6.) as a training dataset and verifying dataset, respectively.

In the training group, *CD38* has 0.960, 0.976, and 0.829 of AUC in three comparing groups (C vs EA, C vs LA, EA vs LA) respectively and *CDKN1C* has 0.852, 0.868 and 0.955 of AUC in three comparing groups (C vs EA, C vs LA, EA vs LA) respectively (Fig. 5D–F). In the verifying group, the staging diagnostic value of *CD38* and *CDKN1C* in staging for Dengue is as follows (Fig. 5G–I): *CD38* (AUC: 0.897, C vs EA; AUC: 0.957, C vs LA; AUC: 0.827, EA vs LA), *CDKN1C* (AUC: 0.575, C vs EA; AUC: 0.929 C vs LA; AUC: 0.876, EA vs LA). Figure 5J–L show high diagnostic values in the testing dataset: *CD38* (AUC: 0.822, C vs EA; AUC: 0.996 C vs LA; AUC: 0.926, EA vs LA), *CDKN1C* (AUC: 0.744, C vs EA; AUC: 0.724 C vs LA; AUC: 0.842, EA vs LA). The analytical results above show that *CD38* can distinguish admirably different phases for Dengue patients and has a higher distinguishable value than *CDKN1C*. Although we consider serotypes (serotype I–IV), results are similar among three comparison groups (C vs EA, C vs LA, EA vs LA) (Additional file 2: Figure S2, Additional file 3: Figure S3).

In addition, as Fig. 5C shown, *CD38* distinguishes perfectly Dengue samples from normal samples with an AUC of 100% in GSE51808 dataset (9 normal samples and 28 Dengue samples). Therefore, *CD38* shows the high value in distinguishing stages for Dengue patients. We selected *CD38* as a biomarker for staging diagnosis of Dengue.

There is no significant difference in expression levels of *CD38* and *CDKN1C* between DH and DHF samples (Additional file 1: Figure S1 L), and different serotypes of Dengue also do not show obvious difference in expression levels of *CD38* and *CDKN1C* (Additional file 1: Figure S1 M).

### Exploration of biomarkers for DHF

**Fig. 6:**
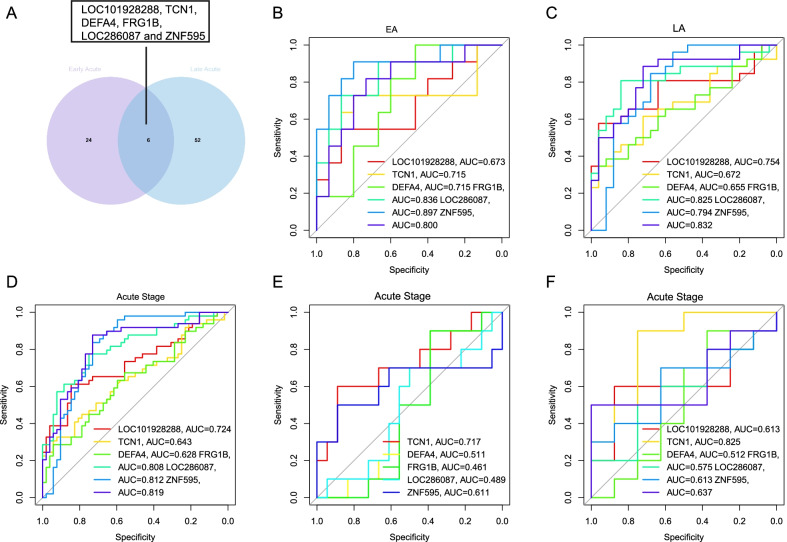
**A** Shared differentially expressed genes (DEGs) between Dengue Fever (DF) and Dengue Hemorrhagic Fever (DHF) in the Acute stage. Analyzing diagnosis value of *LOC101928288*, *TCN1*, *DEFA4*, *FRG1B, LOC286087* and *ZNF595* for Dengue severity by Area Under the Curve (AUC): **B–C** in the training dataset, **D** in the verifying dataset, **E–F** in testing datasets. (C, Convalescent stage; EA, Early Acute stage; LA, Late Acute stage)

After intersecting 30 DEGs between DF (n = 15) and DHF (n = 11) samples from GSE43777 dataset on GLP570 platform in the EA stage with 58 DEGs between DF (n = 25) and DHF (n = 26) samples in the LA stage, we identify 6 shared DEGs (*LOC101928288*, *TCN1*, *DEFA4*, *FRG1B, LOC286087* and *ZNF595*) (Fig. 6A). We speculate that *LOC101928288*, *TCN1*, *DEFA4*, *FRG1B*, *LOC286087* and *ZNF595* can be used as biomarkers to discriminate DHF patients from DF patients due to different expression of them between DHF and DF. The GSE43777 dataset analyze on the GPL570 platform in the EA stage (DF = 15; DHF = 11) (Fig. 6B) and in the LA stage (DF = 25; DHF = 26) (Fig. 6C) are regarded as a training dataset; GSE43777 dataset analyzed on the GPL201 platform in Acute stage (DF = 40; DHF = 37) is regarded as a verifying dataset; GSE51808 (DF = 18; DHF = 10) and GSE18090 (DF = 8; DHF = 10) datasets are regarded as testing datasets. The ROC results (Fig. 6B–F) show *ZNF595* always has acceptable diagnostic value in distinguishing DHF patients from DF patients. Therefore, *ZNF595* is selected as a biomarker to predict DHF patients in Acute stages.

### Immune landscape related to characteristics of dengue patients

GO enrichment analyses for 58 DEGs between DHF samples and DF samples show neutrophil and humoral immune response are activated and KEGG pathway enrichment analyses show rich inflammatory pathways. Therefore, we analyze fraction changes for 22 types of immune cells during DENV infection by CIBERSORT. The GSE43777 dataset analyze on the GLP201 platform is used to explore fractions of 22 types of immune cells in whole blood samples from Dengue patients in three phases and the GSE43777 dataset analyzed on the GLP570 platform is applied to explore differently infiltrating-immune characteristic between DF and DHF samples.

**Fig. 7:**
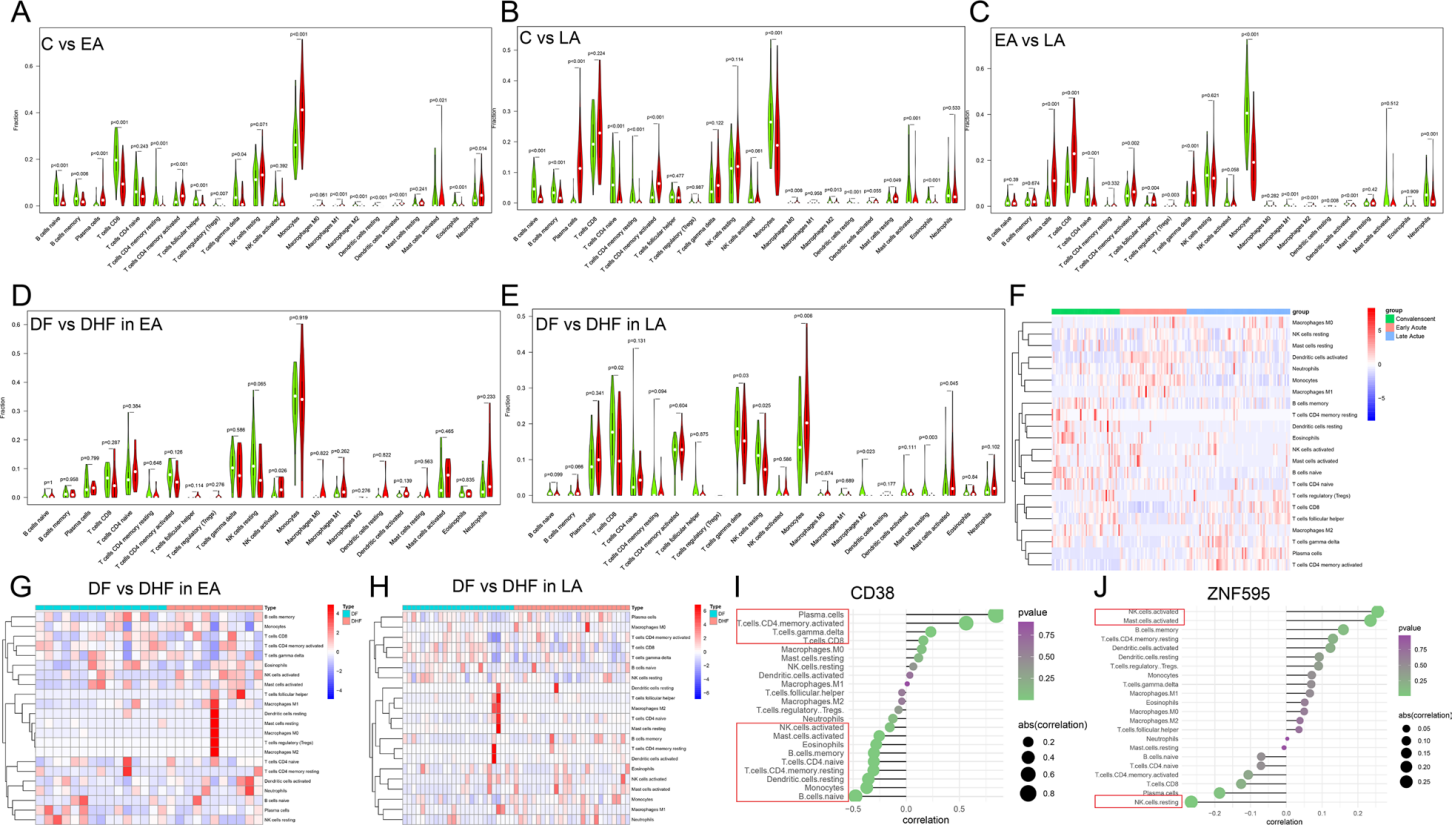
**A–E** Violin diagrams and **F–H** heatmaps display immune differences of immune cells in different comparing groups. **I–J** Correlation between genes (*CD38* and *ZNF595*) and 22 types of immune cells (red rectangular: statistically significant (*P* < 0.05)). (C, Convalescent stage; EA, Early Acute stage; LA, Late Acute stage)

As Fig. 7A–C and 7F shown, in the EA phase, fractions of activated dendritic cells, Neutrophils, Monocytes, and M1 Macrophages (*P* < 0.05) increase significantly compared to the LA and C phases. Compared the LA phase with the EA phase and the C phase, fractions of Plasma cells and activated memory CD4+ T cells (*P* < 0.05) increase clearly (Fig. 7A–C and F). While Compared with the EA phase and the LA phase, the increments in fractions of memory B cells, resting memory CD4+ T cells, resting dendritic cells, Eosinophils, and naïve B cells (*P* < 0.05) are more obvious in the C phase (Fig. 7A–C and F). Analytical results above mean that immune cells with antigen presentation, phagocytosis and chemotaxis, immune cells involved in humoral immunity and memory cells are more obviously active in the EA, LA and C phase, respectively. When analyzing in a single serotype, we can still get above results (Additional file 4: Figure S4).

In the LA stage, fractions of activated NK cells (*P* < 0.05) increase in DHF samples (Fig. 7E and H), but fractions of CD8+ T cells, gamma delta T cells, resting NK cells, M2 Macrophages and resting mast cells decrease (Fig. 7E and H) (*P* < 0.05), which imply immune response are damaged in DHF samples. In the EA stage, the immune response of DF patients is similar to that of DHF patients (Fig. 7D and G). The immune response of DHF patients in the LA stage is significantly impaired which explain rapid deterioration of DHF patients in the LA stage.

### Correlations between gene and immune cells

We analyze correlations between *CD38* and immune cells in GSE43777 dataset (Fig. 7I). The infiltration level of Plasma cells, activated memory CD4+ T cells, gamma delta T cells and CD8+ T cells are positively related to *CD38* (Fig. 7I); the infiltration level of naïve B cells, Monocytes, resting dendritic cells, resting memory CD4+ T cells, naïve CD4+ T cells, memory B cells, Eosinophils and activated mast cells are negatively related to *CD38* (Fig. 7I). Therefore, the analytical results above show there is strong co-expression relationship between *CD38* and plasma cells and between *CD38* and activated memory CD4+ T cells (Pearson’s correlation > 0.5). In individual serotype, we can still draw this conclusion (Additional file 5: Figure S5). Correlation between immune cells and *ZNF595* is not significant (Fig. 7J).

### Analyzing the staging characteristic of immune cells

**Fig. 8:**
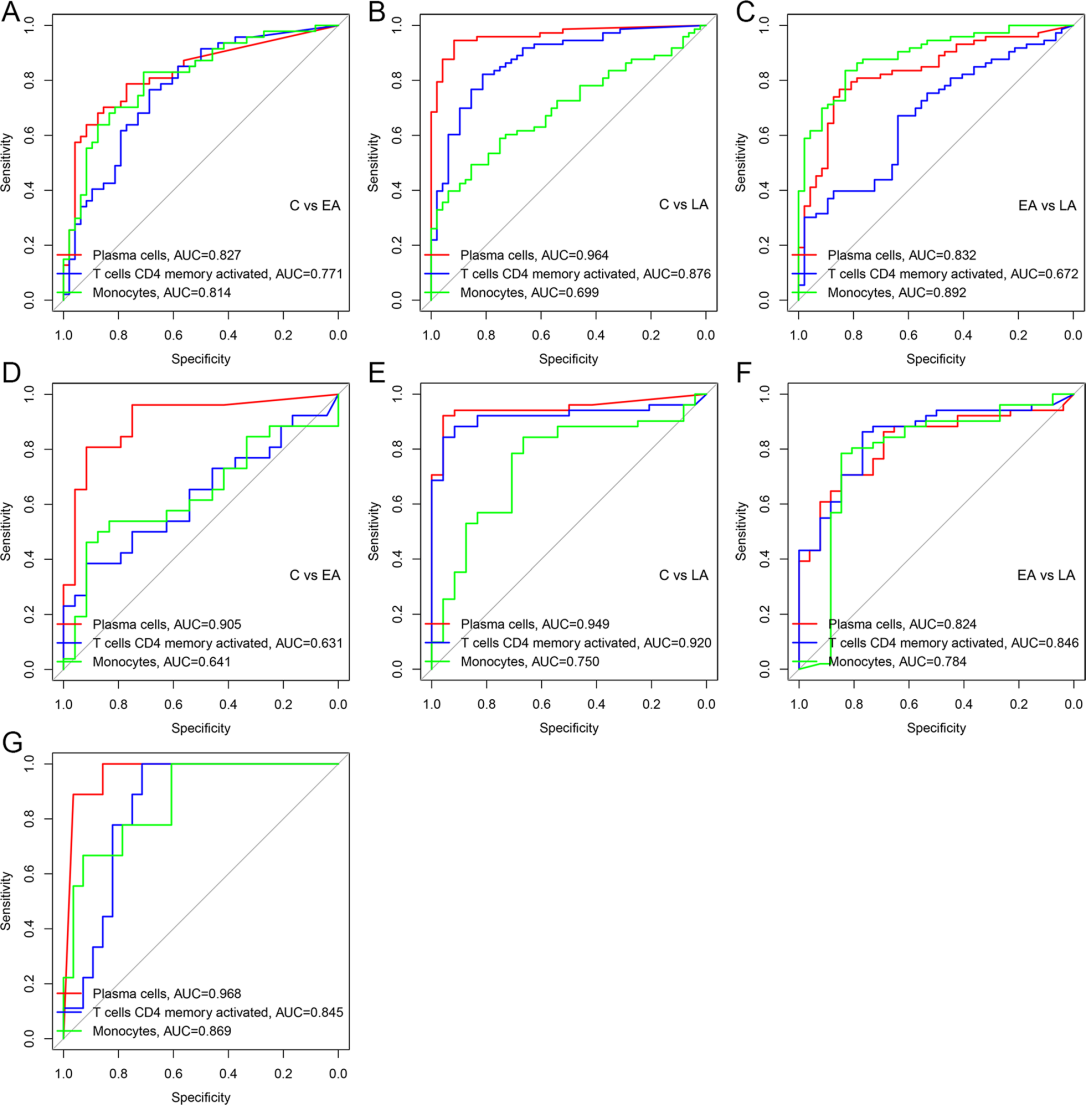
Analyzing staging diagnosis value of immune cells for Dengue by Area Under the Curve (AUC). **A–C** in the training group and **D–F** in the test group. **G **Validating diagnosis value of immune cells between Dengue samples and normal samples. (C, Convalescent stage; EA, Early Acute stage; LA, Late Acute stage)

Because of high co-expression correlation between *CD38* and plasma cells, we speculated that infiltrating-immune plasma cells could also show distinct differences in three stages. To explore this characteristic, we regarded GSE43777 analyzed by GPL201 as a training set and GSE43777 analyzed by GPL570 as a test set. As the Fig. 8A–C shown in the training group, the distinguishing value of Plasma cells, activated memory CD4+ T cells, and Monocytes in staging were good and Plasma cells have the highest distinguishing value (AUC:0.827, C vs EA; AUC:0.964, C vs LA; AUC: 0.832, EA vs LA). In the test group, the distinguishing value of three types of immune cells in stage for Dengue was still valuable and Plasma cells have the highest distinguishing value in Fig. 8D–F (AUC: 0.905, C vs EA; AUC: 0.949, C vs LA; AUC: 0.824, EA vs LA. Plasma cells (AUC = 0.968), activated memory CD4 + T cells (AUC = 0.845) and Monocytes (AUC = 0.869) can excellently distinguish Dengue samples from normal samples in GSE51808 (Fig. 8G). When considering different serotypes, above results were still obtained (Additional file 6: Figure S6). Therefore, we can discriminate three stages based on the fraction of Plasma cells, activated memory CD4 + T cells and Monocytes in Dengue patients.

## Discussion

This study combined DEGs analysis, WGCNA and ROC to identify, valid and test potential biomarkers associated with the staging and severity of Dengue, and used GO enrichment analysis, KEGG analysis and GSEA analysis to explore potential reasons resulting in DHF. The CIBERSORT website was also applied to explore immune differences during Dengue infection. Our research was the first to show that *CD38* and *ZNF595* had clinical significance in the stage and severity of Dengue and that they could be used as biomarkers to distinguish clinical stages and severity for Dengue patients. It was worth noting that Plasma cells, activated memory CD4+ T cells, and Monocytes also showed distinguishing value.

*CD38* which was identified, verified and tested in independent datasets could distinguish three clinical stages of Dengue fever and was significantly associated with plasma cells, but it could not use to predict severity which was similar to precious results [52] and distinguish different serotypes. Our results showed expression levels of *CD38* and fractions  of plasma cells were similar in four serotypes. We identified, verified and tested *ZNF595* in independent datasets that could predict DHF. Mechanistically, EA-DEGs which inhibited viral replication were down-regulated in DHF, and a related-autophagy gene (*CCL2*) expressed differently and increased significantly in DHF, all of which suggested DENV regulated autophagy to lead to DHF.

In addition, we analyzed systematically immune differences among three stages and between DHF and DF in the acute stage, and correlation between immune cells and genes. Immune analysis results showed fractions of monocytes, activated mast cells, M1 macrophages and neutrophils referred to innate immune response increased obviously in the EA stage compared with the C stage; significant increments can be observed in fractions of plasma cells and activated memory CD4+ T cells comparing the LA stage with the C stage; increasing fractions of plasma cells, CD8+ T cells, activated memory CD4+ T cells, follicular helper T cells, regulatory T cells (Tregs) and gamma delta T cells can be discovered in the LA stage compared with the EA stage. Above results were suitable for any serotype, suggesting different serotypes did not have obvious immune differences. Increasing immune response eliminated DENV which avoided Dengue symptoms [27] and immune response kinetics dependent on initial lymphocyte numbers, were observed to correspond with illness severity [53]. Our studies showed neutrophil and humoral immune response are activated in DHF in the LA stage, but the whole immune system was damaged in DHF compared with DF, which was a possible reason leading to DHF.

Interestingly, KEGG enrichment showed DEGs (between the EA stage and the C stage, and between the EA stage and the LA stage) were enriched in Coronavirus disease —COVID-19, Influenza A, Hepatitis C and Measles pathways, implying Coronavirus [54], Alphainfluenzavirus influenzae [55], Hepatitis C virus [56] and Measles morbillivirus also up regulated these genes during infection and had shared pathogenic mechanism.

Gene expression profiles from public databases (GEO) have been applied to explore Dengue biomarkers by researchers. Several studies have used multiple genes to distinguish Dengue patients from healthy samples [39, 40], DHF from DF [41] and different stages [57]. However, multi-gene lists can limit the sensitivity and specificity of DEGs as disease biomarkers [58, 59].

Compared with previous studies [39–41], our study had several advantages. First of all, this study was based on single gene analyses which allowed us to identify a stable and robust biomarker and these biomarkers were identified and verified on independent datasets which increased the accuracy of our study. Secondly, we combined three methods, including DEG, WGCNA and ROC to screen, verify and test biomarkers for Dengue diagnoses which increase the accuracy of our results, differing from precious studies only depending on the DEG analysis. Thirdly, we analyzed 22 types of immune cells including less attention immune cells based on RNA-sequence which contributed to increase understand of the whole immune response during DENV infection and explore correlations between genes and immune cells. A previous study showed that peripheral lymphocyte subset alteration can be independent predictors for clinical characteristics and treatment efficacy of COVID-19 [60]. Interestingly, we found that the fraction of Plasma cells, activated memory CD4+ T cells and Monocytes in Dengue patients also had clinical characteristics and can distinguish these clinical stages for Dengue patients. Finally, not just for single serotype, our study included four serotypes which was involved in separate and combined analysis.

There is still a shortcoming of this study, because the study only uses public datasets to analyze, verify and test, without clinical verification and test. Our research has provided help in distinguishing the stage and severity of Dengue infection and understanding pathogenic mechanism of different serotypes, and will help analyze mechanisms of DHF and benefit to clinical treatment in the future.

## Conclusions

In conclusion, based on expression levels of *CD38* and fractions of Plasma cells, activated memory CD4+ T cells and Monocytes, we can discriminate admirably three clinical stages for Dengue patients (results are suitable for any serotype); *ZNF595* can better distinguish dengue hemorrhagic fever (DHF) from Dengue fever (DF). Up-regulated autophagy-related genes contribute to understand mechanisms of DHF.

## Supplementary Information

**Additional file 1. Figure S1.** (**A**–**C**) PCA shows that different stages can be distinguished. Weighted co-expression network analysis (WGCNA). (**D**–**F**) Showing the cutoff height. (**G**–**I**) Sample clustering. (J) Shared gene (*CCL2*) in the LA stage between DEGs (between Dengue Hemorrhagic Fever (DHF) and Dengue Fever (DF)) and 232 autophagy-related genes, and (**K**) its expression levels. Similar expression levels of *CD38* and *CDKN1C* between DH and DHF (**L**) and among different serotypes (**M**). S1, serotype I; S2, serotype II; S3, serotype III; S4, serotype IV. (C, Convalescent stage; EA, Early Acute stage; LA, Late Acute stage).

**Additional file 2. Figure S2.** Analyzing staging diagnosis value of *CD38* and *CDKN1C* for four serotypes among three comparison groups by Area Under the Curve (AUC) in the training set (GSE43777 dataset of GLP201 platform), respectively. (**A**) C vs EA in serotype I; (**B**) C vs LA in serotype I; (**C**) EA vs LA in serotype I; (**D**) C vs EA in serotype II; (**E**) C vs LA in serotype II; (**F**) EA vs LA in serotype II; (**G**) C vs EA in serotype III; (**H**) C vs LA in serotype III; (**I**) EA vs LA in serotype III; (**J**) C vs EA in serotype IV; (**K**) C vs LA in serotype IV; (**L**) EA vs LA in serotype IV. (C, Convalescent stages; EA, Early Acute stage; LA, Late Acute stage).

**Additional file 3. Figure S3.** Analyzing staging diagnosis value of *CD38* and *CDKN1C* for four serotypes among three comparison groups by Area Under the Curve (AUC) in the test set (GSE43777 dataset of GLP570 platform), respectively. (**A**) C vs EA in serotype I; (**B**) C vs LA in serotype I; (**C**) EA vs LA in serotype I; (**D**) C vs EA in serotype II; (**E**) C vs LA in serotype II; (**F**) EA vs LA in serotype II; (**G**) C vs EA in serotype III; (**H**) C vs LA in serotype III; (**I**) EA vs LA in serotype III; (**J**) C vs EA in serotype IV; (**K**) C vs LA in serotype IV; (**L**) EA vs LA in serotype IV. (C, Convalescent stage; EA, Early Acute stage; LA, Late Acute stage).

**Additional file 4. Figure S4.** Violin diagrams and heatmaps display immune differences of immune cells in different comparing groups. (**A**) C vs EA stages in the serotype I. (**B**) C vs LA stages in the serotype I. (**C**) EA vs LA stages in the serotype I. (**D**) Immune difference heatmap of the serotype I in three stages. (**E**) C vs EA stages in the serotype II. (**F**) C vs LA stages in the serotype II. (**G**) EA vs LA stages in the serotype II. (**H**) Immune difference heatmap of the serotype II in three stages. (**I**) C vs EA stages in the serotype III. (**J**) C vs LA stages in the serotype III. (**K**) EA vs LA stages in the serotype III. (**L**) Immune difference heatmap of the serotype III in three stages. (**M**) C vs EA stages in the serotype IV. (**N**) C vs LA stages in the serotype IV. (**O**) EA vs LA stages in the serotype IV. (**P**) Immune difference heatmap of the serotype IV in three stages. (C, Convalescent stage; EA, Early Acute stage; LA, Late Acute stage).

**Additional file 5. Figure S5. ** Correlation between *CD38* and 22 types of immune cells (red rectangular: statistically significant (P < 0.05)). (**A**) In serotype I; (**B**) in serotype II; (**C**) in serotype III; (**D**) in serotype IV. (C, Convalescent stage; EA, Early Acute stage; LA, Late Acute stage).

**Additional file 6. Figure S6.** Analyzing staging diagnosis value of immune cells for Dengue by Area Under the Curve (AUC) in each serotype. (A) C vs EA stages in the serotype I. (B) C vs LA stages in the serotype I. (C) EA vs LA stages in the serotype I. (D) C vs EA stages in the serotype II. (E) C vs LA stages in the serotype II. (F) EA vs LA stages in the serotype II. (G) C vs EA stages in the serotype III. (H) C vs LA stages in the serotype III. (I) EA vs LA stages in the serotype III. (J) C vs EA stages in the serotype IV. (K) C vs LA stages in the serotype IV. (L) EA vs LA stages in the serotype IV. (C, Convalescent stage; EA, Early Acute stage; LA, Late Acute stage).

**Additional file 7. Table S1.** Differentially expressed genes (DEGs) in the C vs EA group. (C, Convalescent stage; EA, Early Acute stage).

**Additional file 8. Table S2.** Differentially expressed genes (DEGs) in the C vs LA group. (C, Convalescent stage; LA, Late Acute stage).

**Additional file 9. Table S3.** Differentially expressed genes (DEGs) in the EA vs LA group. (EA, Early Acute stage; LA, Late Acute stage).

**Additional file 10. Table S4**. Differentially expressed genes (DEGs) from comparing Dengue Hemorrhagic Fever (DHF) with Dengue Fever (DF) in EA group. (EA, Early Acute stage).

**Additional file 11. Table S5**. Differentially expressed genes (DEGs) from comparing Dengue Hemorrhagic Fever (DHF) with Dengue Fever (DF) in LA group. (LA, Late Acute stage).

**Additional file 12. Table S6**. Differentially expressed fold of genes in individual serotype for three comparison groups. (C, Convalescent stage; EA, Early Acute stage; LA, Late Acute stage).

## Data Availability

The datasets supporting the conclusions of this article are available in the Gene Expression Omnibus (GEO) database (GSE43777, GSE51808 and GSE28405).

## Abbreviations

DEGs: Differentially expressed genes
WGCNA: Weighted co-expression network analysis
ROC: Receiver operator characteristic curve
GO: Gene Ontology
KEGG: Kyoto Encyclopedia of Genes and Genomes
GSEA: Gene set enrichment analysis
AUC: Area under the curve
DHF: Dengue hemorrhagic fever
DF: Dengue fever
WHO: World Health Organization WHO
ADE: Antibody-dependent enhancement
DENV: Dengue virus
MAVS: Mitochondria antiviral protein
DSS: Dengue shock syndrome
PCA: Principal component analysis
C: Convalescent stage
EA: Early acute stage
LA: Late acute stage
NCBI: National Center of Biotechnology Information
PBMC: Peripheral blood mononuclear cell
TOM: Topological overlap matrix
