# Supplementary figures and images for: Identification of stage-related and severity-related biomarkers and exploration of immune landscape for Dengue by comprehensive analyses
Source: Virol J. 2022 Aug 2;19:130. doi: 10.1186/s12985-022-01853-8 (PMC9344228; doi:10.1186/s12985-022-01853-8)

A

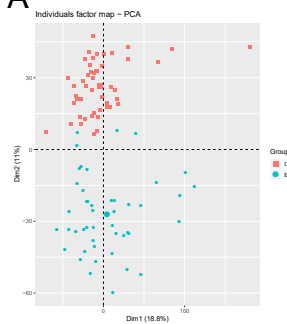

B

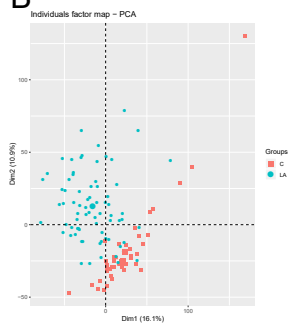

C

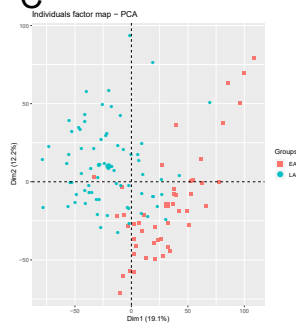

J

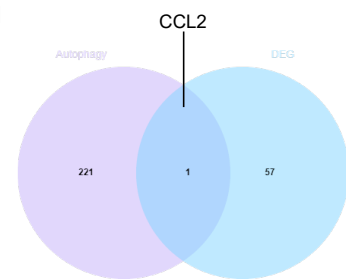

D

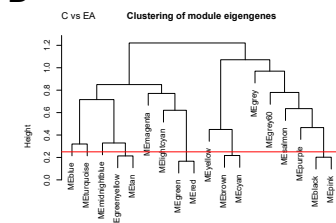

E

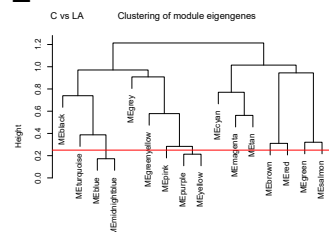

F

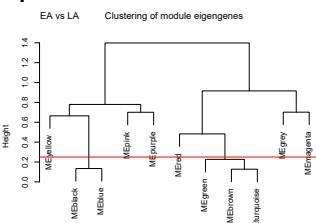

K

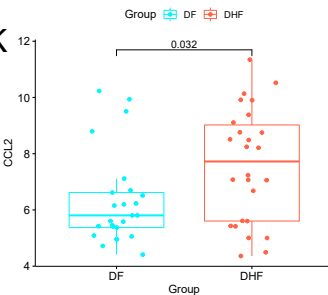

G

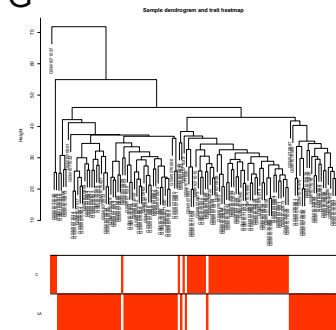

H

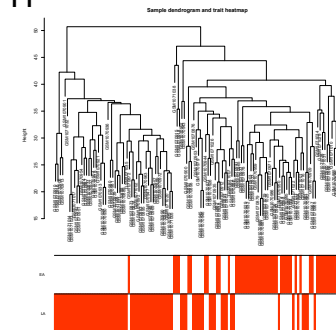

I

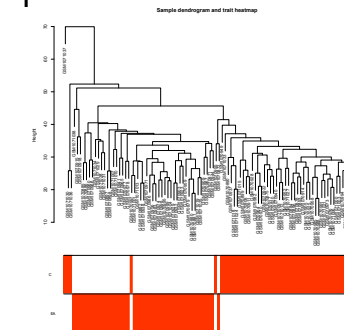

L

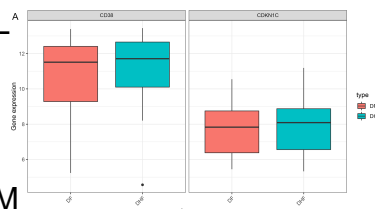

M

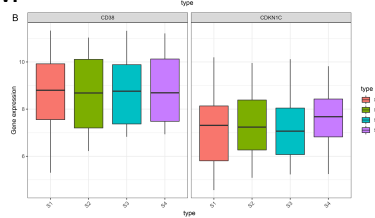

Supplement: Supplementary file 1 — Additional file 1. Figure S1. (A–C) PCA shows that different stages can be distinguished. Weighted co-expression network analysis (WGCNA). (D–F) Showing the cutoff height. (G–I) Sample clustering. (J) Shared gene (CCL2) in the LA stage between DEGs (between Dengue Hemorrhagic Fever (DHF) and Dengue Fever (DF)) and 232 autophagy-related genes, and (K) its expression levels. Similar expression levels of CD38 and CDKN1C between DH and DHF (L) and among different serotypes (M). S1, serotype I; S2, serotype II; S3, serotype III; S4, serotype IV. (C, Convalescent stage; EA, Early Acute stage; LA, Late Acute stage). [file 12985_2022_1853_MOESM1_ESM.pdf]

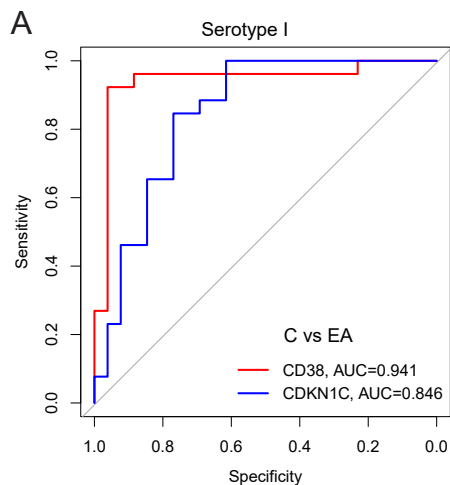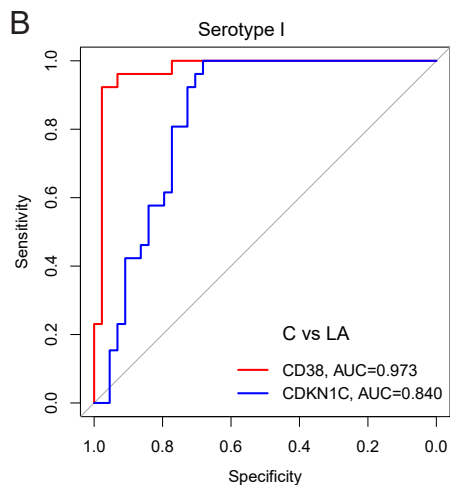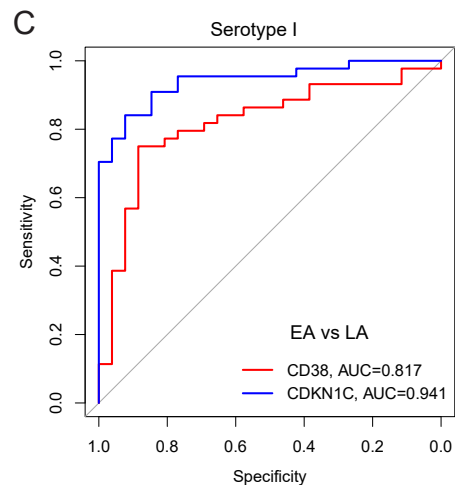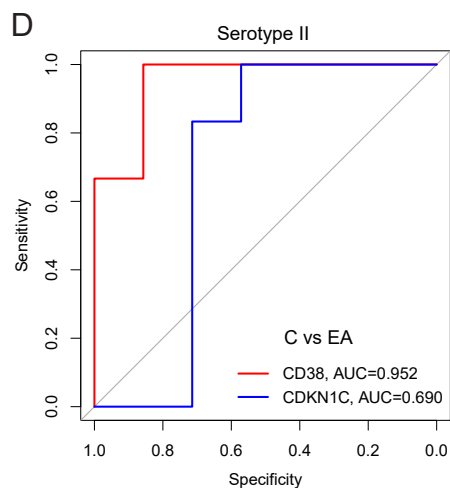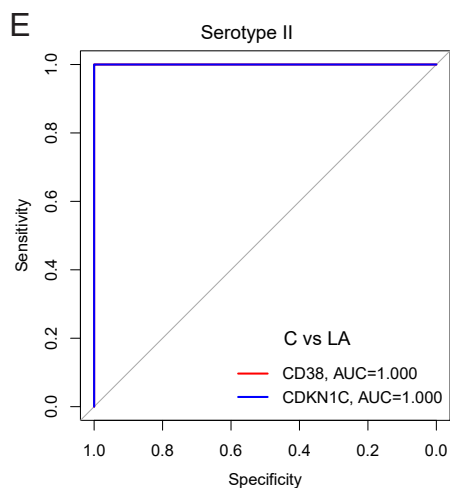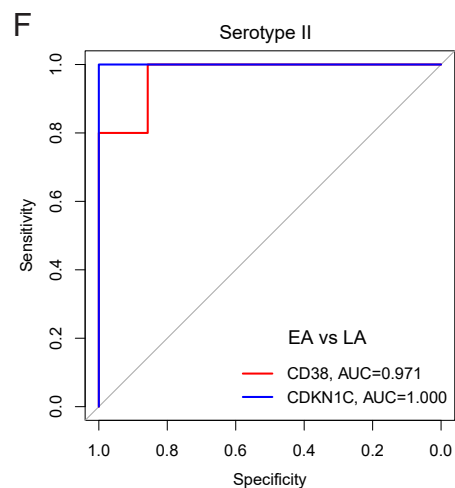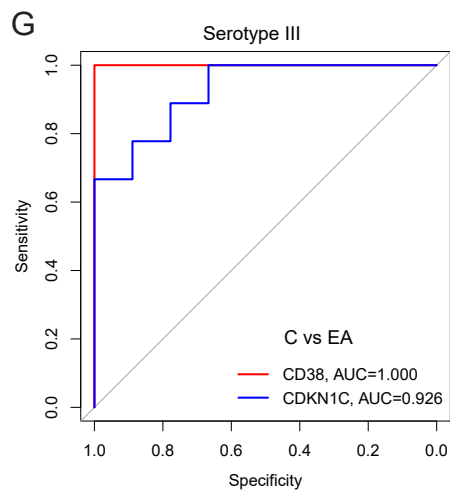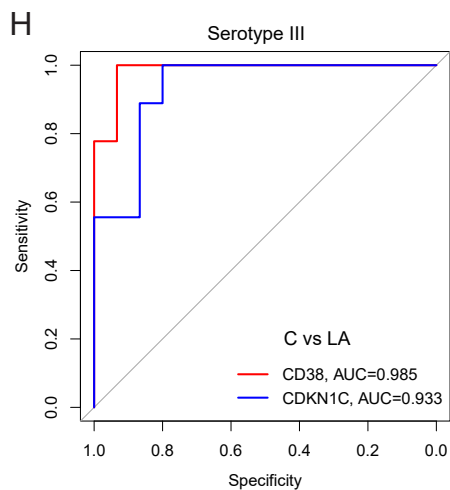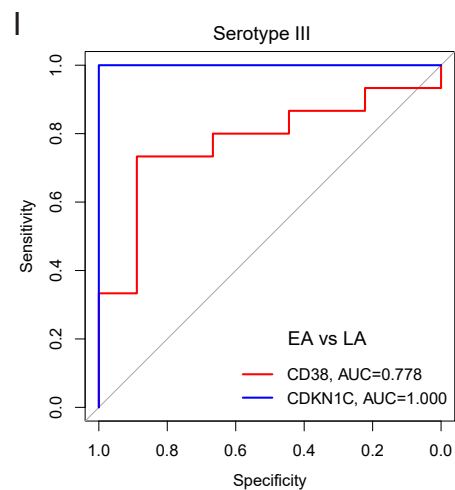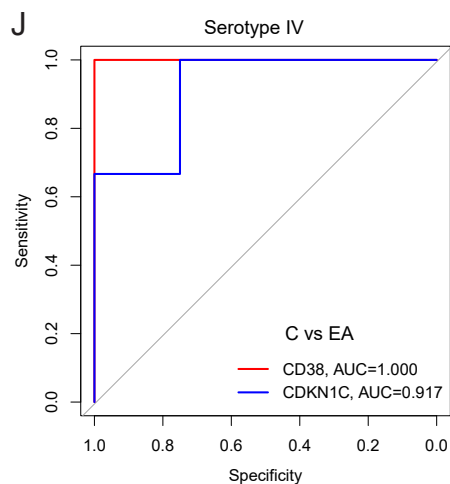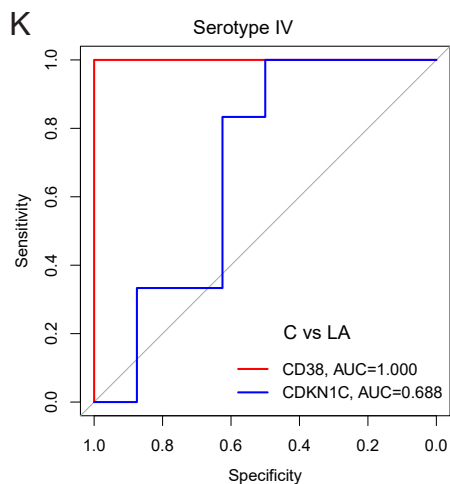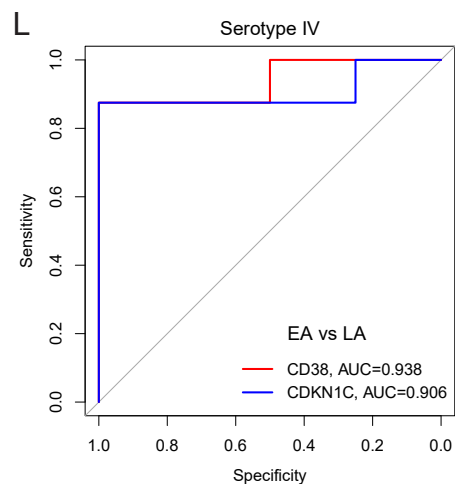

Supplement: Supplementary file 2 — Additional file 2. Figure S2. Analyzing staging diagnosis value of CD38 and CDKN1C for four serotypes among three comparison groups by Area Under the Curve (AUC) in the training set (GSE43777 dataset of GLP201 platform), respectively. (A) C vs EA in serotype I; (B) C vs LA in serotype I; (C) EA vs LA in serotype I; (D) C vs EA in serotype II; (E) C vs LA in serotype II; (F) EA vs LA in serotype II; (G) C vs EA in serotype III; (H) C vs LA in serotype III; (I) EA vs LA in serotype III; (J) C vs EA in serotype IV; (K) C vs LA in serotype IV; (L) EA vs LA in serotype IV. (C, Convalescent stages; EA, Early Acute stage; LA, Late Acute stage). [file 12985_2022_1853_MOESM2_ESM.pdf]

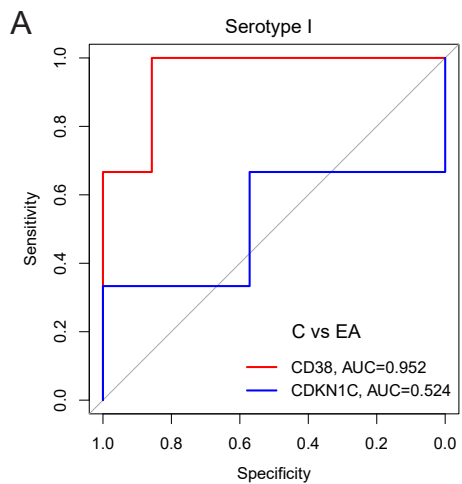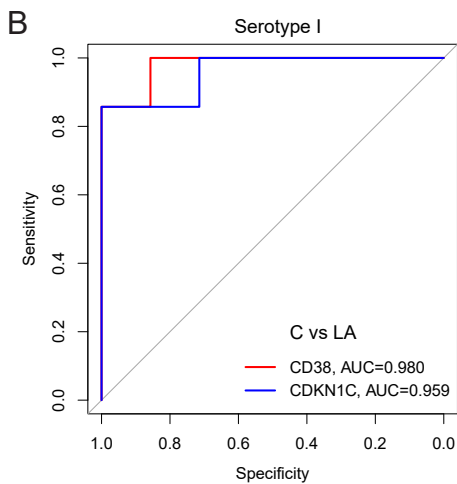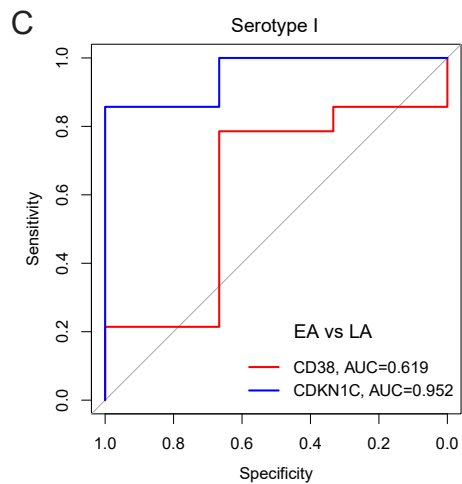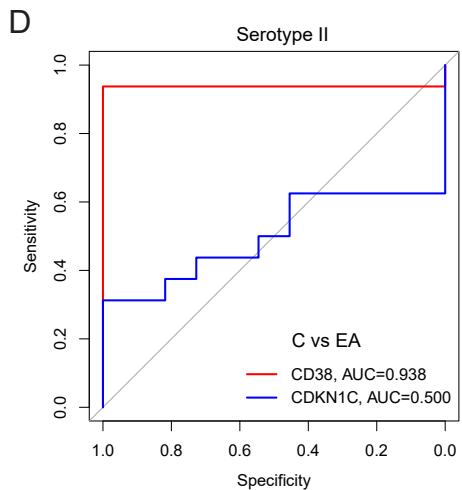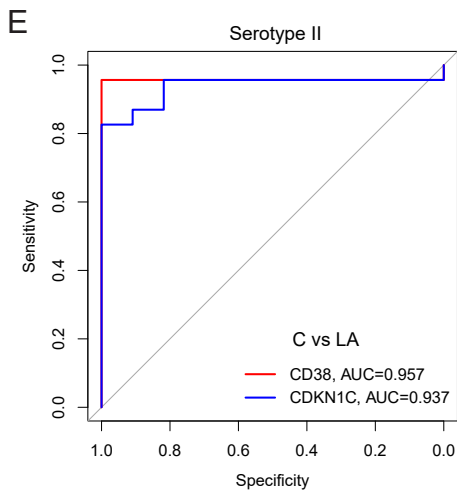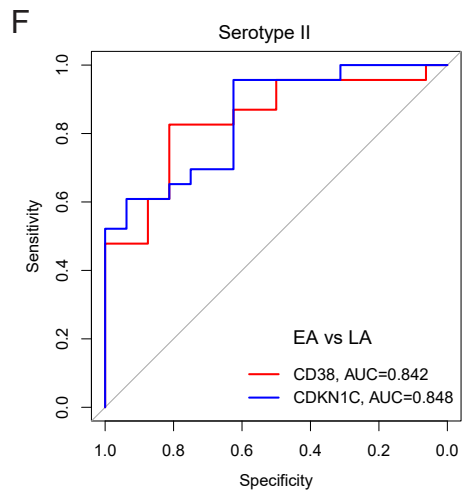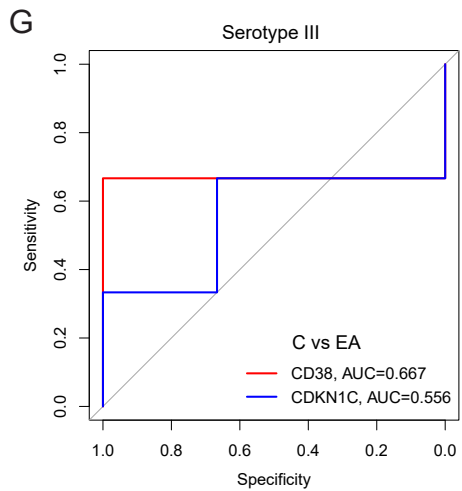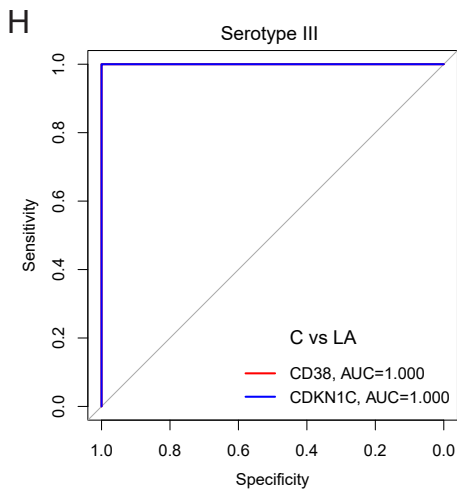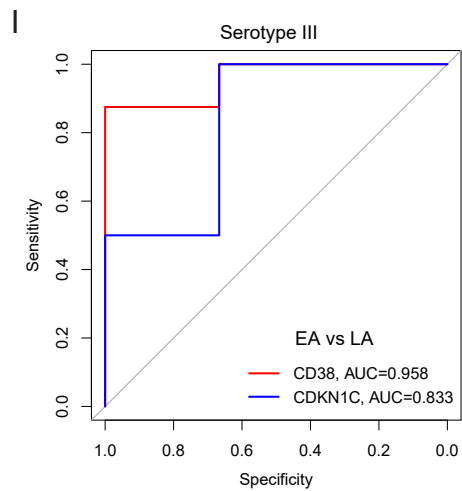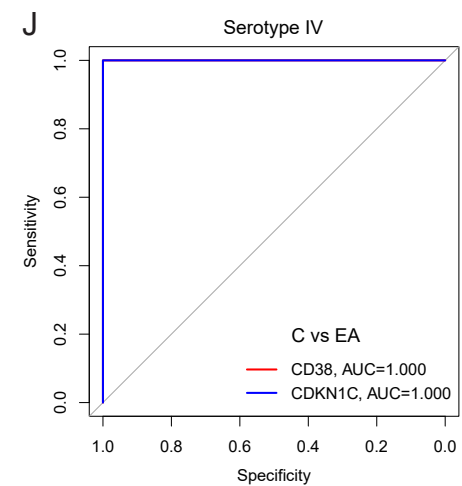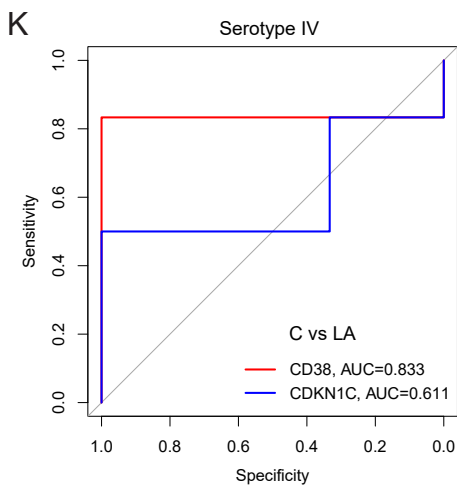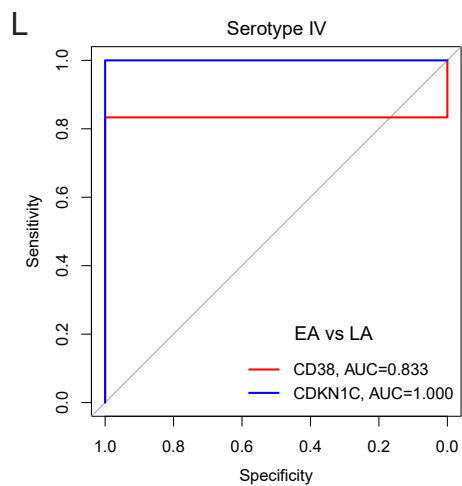

Supplement: Supplementary file 3 — Additional file 3. Figure S3. Analyzing staging diagnosis value of CD38 and CDKN1C for four serotypes among three comparison groups by Area Under the Curve (AUC) in the test set (GSE43777 dataset of GLP570 platform), respectively. (A) C vs EA in serotype I; (B) C vs LA in serotype I; (C) EA vs LA in serotype I; (D) C vs EA in serotype II; (E) C vs LA in serotype II; (F) EA vs LA in serotype II; (G) C vs EA in serotype III; (H) C vs LA in serotype III; (I) EA vs LA in serotype III; (J) C vs EA in serotype IV; (K) C vs LA in serotype IV; (L) EA vs LA in serotype IV. (C, Convalescent stage; EA, Early Acute stage; LA, Late Acute stage). [file 12985_2022_1853_MOESM3_ESM.pdf]

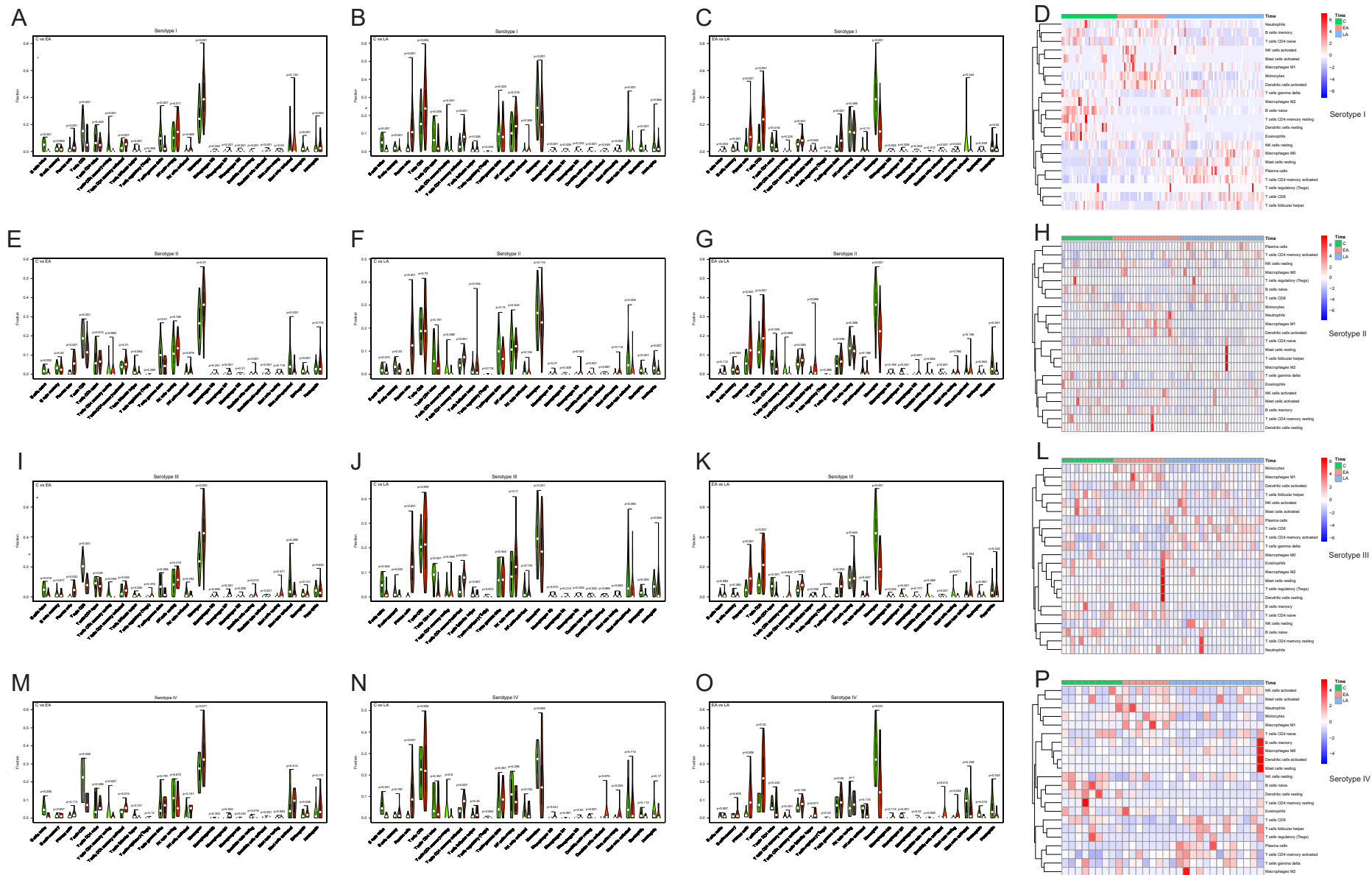

Supplement: Supplementary file 4 — Additional file 4. Figure S4. Violin diagrams and heatmaps display immune differences of immune cells in different comparing groups. (A) C vs EA stages in the serotype I. (B) C vs LA stages in the serotype I. (C) EA vs LA stages in the serotype I. (D) Immune difference heatmap of the serotype I in three stages. (E) C vs EA stages in the serotype II. (F) C vs LA stages in the serotype II. (G) EA vs LA stages in the serotype II. (H) Immune difference heatmap of the serotype II in three stages. (I) C vs EA stages in the serotype III. (J) C vs LA stages in the serotype III. (K) EA vs LA stages in the serotype III. (L) Immune difference heatmap of the serotype III in three stages. (M) C vs EA stages in the serotype IV. (N) C vs LA stages in the serotype IV. (O) EA vs LA stages in the serotype IV. (P) Immune difference heatmap of the serotype IV in three stages. (C, Convalescent stage; EA, Early Acute stage; LA, Late Acute stage). [file 12985_2022_1853_MOESM4_ESM.pdf]

A

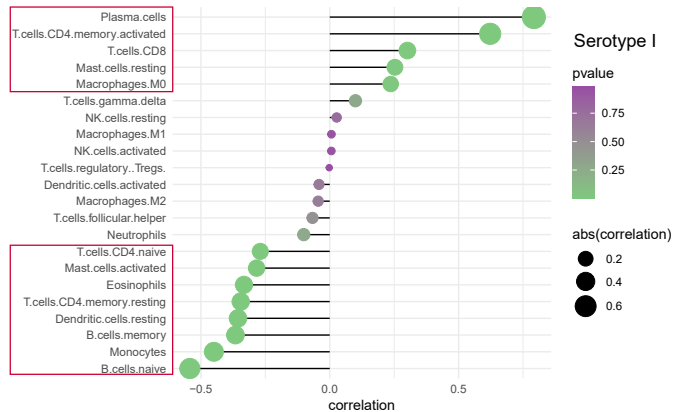

B

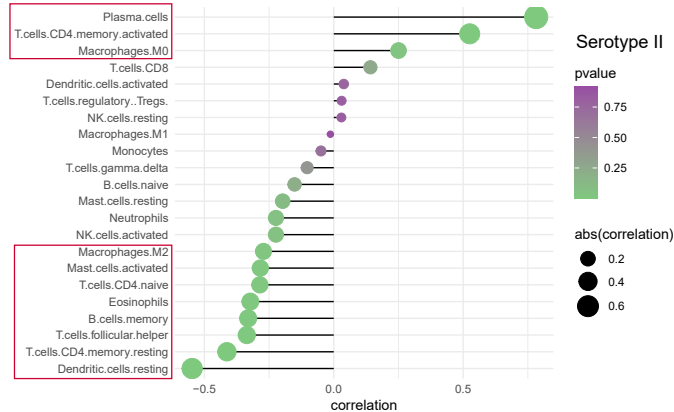

C

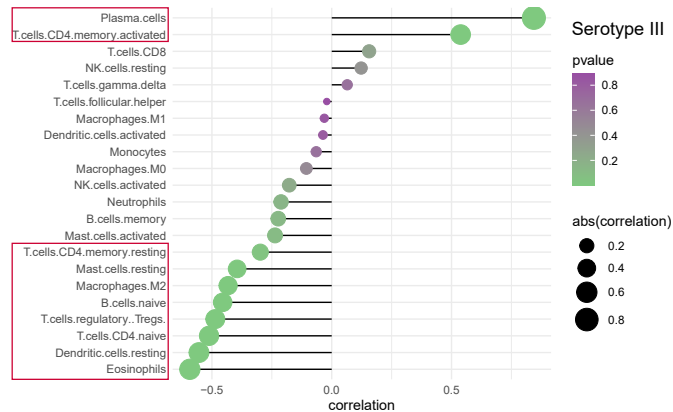

D

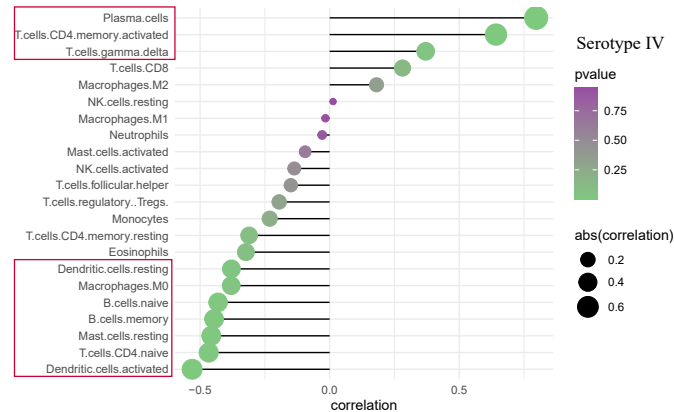

Supplement: Supplementary file 5 — Additional file 5. Figure S5. Correlation between CD38 and 22 types of immune cells (red rectangular: statistically significant (P < 0.05)). (A) In serotype I; (B) in serotype II; (C) in serotype III; (D) in serotype IV. (C, Convalescent stage; EA, Early Acute stage; LA, Late Acute stage). [file 12985_2022_1853_MOESM5_ESM.pdf]

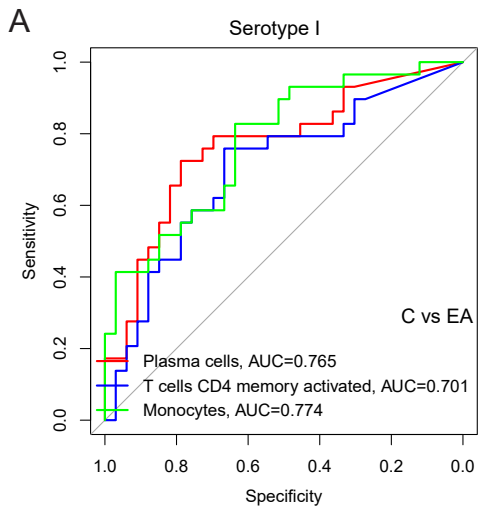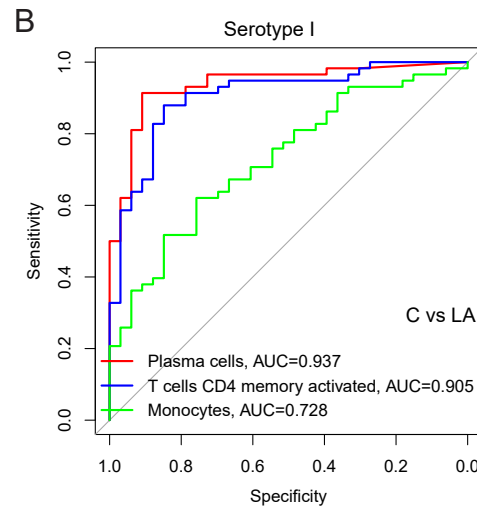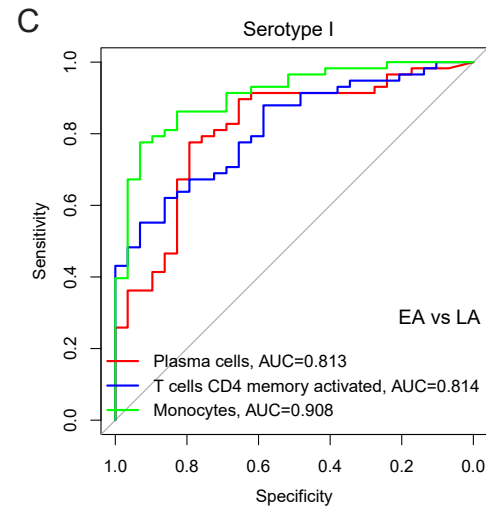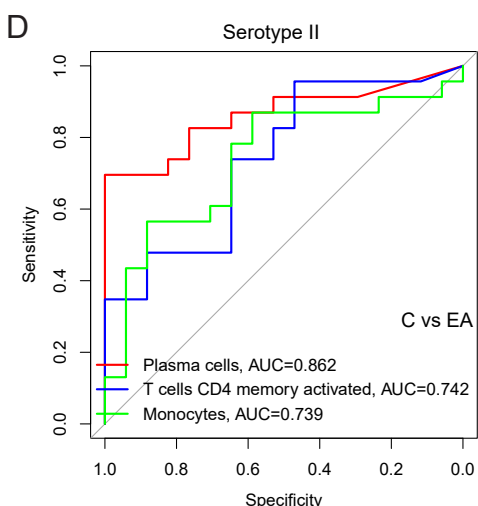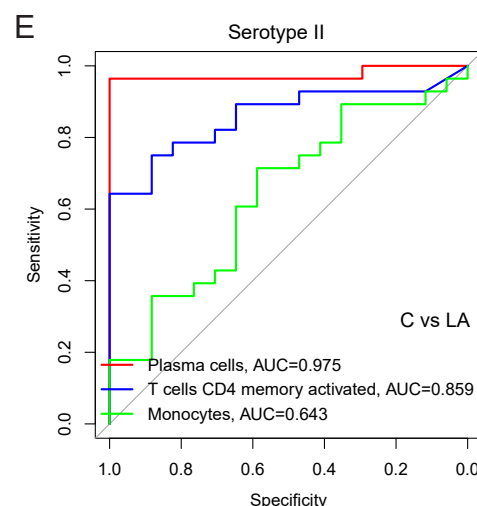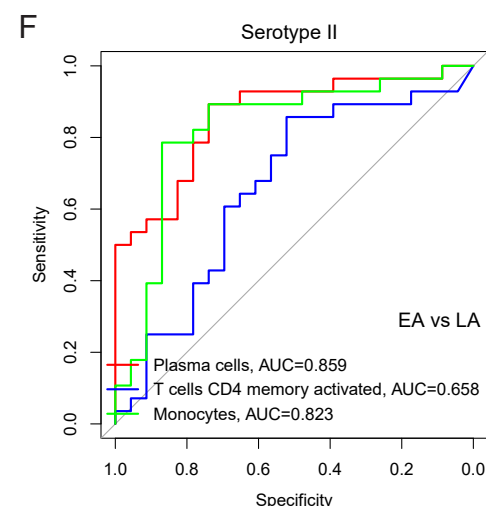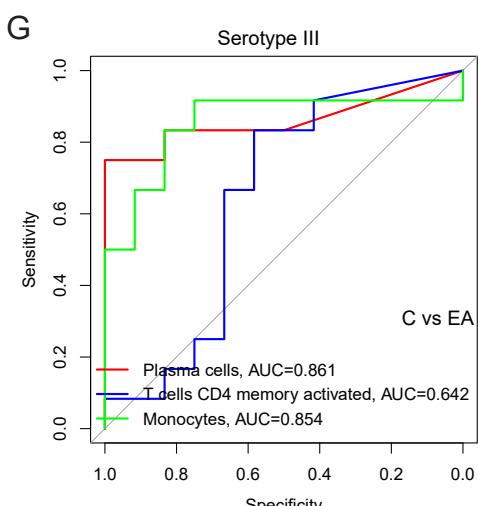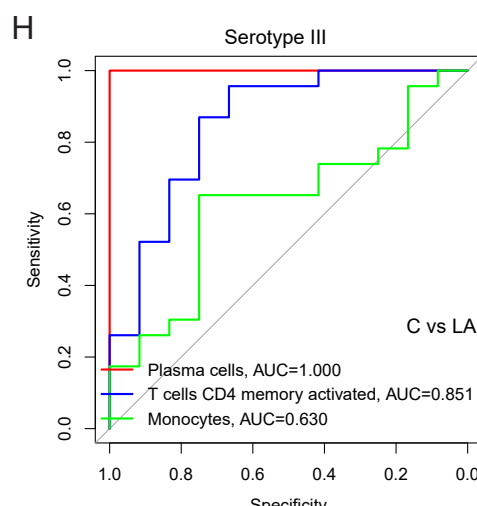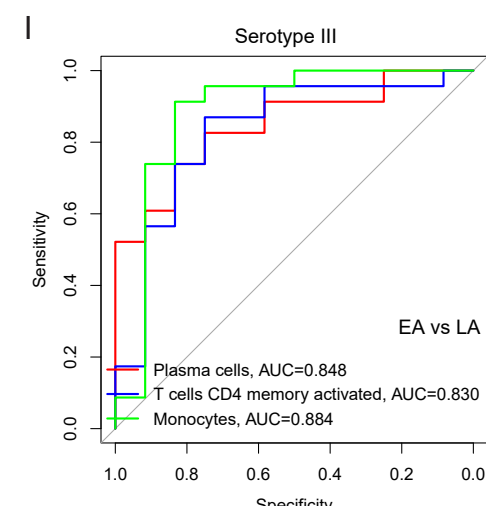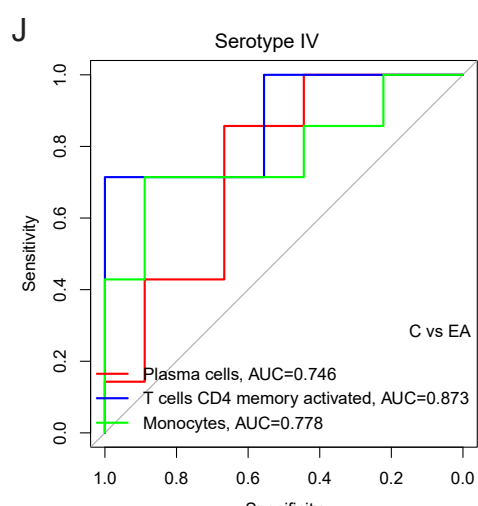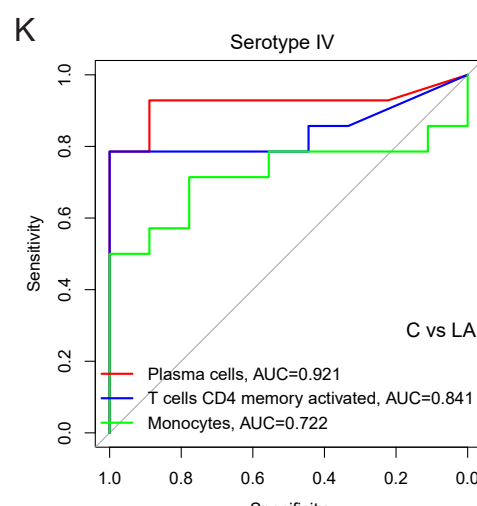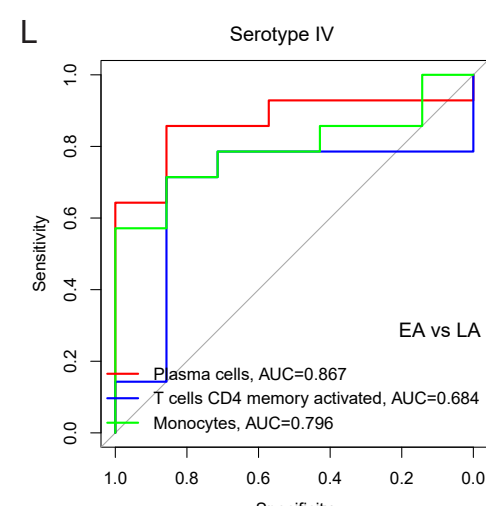

Supplement: Supplementary file 6 — Additional file 6. Figure S6. Analyzing staging diagnosis value of immune cells for Dengue by Area Under the Curve (AUC) in each serotype. (A) C vs EA stages in the serotype I. (B) C vs LA stages in the serotype I. (C) EA vs LA stages in the serotype I. (D) C vs EA stages in the serotype II. (E) C vs LA stages in the serotype II. (F) EA vs LA stages in the serotype II. (G) C vs EA stages in the serotype III. (H) C vs LA stages in the serotype III. (I) EA vs LA stages in the serotype III. (J) C vs EA stages in the serotype IV. (K) C vs LA stages in the serotype IV. (L) EA vs LA stages in the serotype IV. (C, Convalescent stage; EA, Early Acute stage; LA, Late Acute stage). [file 12985_2022_1853_MOESM6_ESM.pdf]
