# Supplementary material for: Identification of stage-related and severity-related biomarkers and exploration of immune landscape for Dengue by comprehensive analyses
Source: Virol J. 2022 Aug 2;19:130. doi: 10.1186/s12985-022-01853-8 (PMC9344228; doi:10.1186/s12985-022-01853-8)
Supplement: Supplementary file 7 — Additional file 7. Table S1. Differentially expressed genes (DEGs) in the C vs EA group. (C, Convalescent stage; EA, Early Acute stage). [file 12985_2022_1853_MOESM7_ESM.pdf]

Gene	logFC	AveExpr	t	P.Value	adj.P.Val
IFI27	6.476419	8.517755	36.01416	1.15E-57	9.30E-54
SERPING1	3.766264	7.435511	31.21745	3.71E-52	1.50E-48
ISG15	3.355613	10.71839	25.21172	3.42E-44	9.21E-41
MX1	2.858691	10.62336	24.13974	1.27E-42	2.57E-39
IFITM3	1.993669	12.05478	23.70347	5.73E-42	9.27E-39
IFITM1	1.455452	12.0561	23.24421	2.86E-41	3.85E-38
USP18	2.562914	6.910707	22.98036	7.26E-41	8.39E-38
IRF7	2.510148	9.172833	22.92098	8.97E-41	9.06E-38
LGALS3BP	2.31518	7.826941	22.52306	3.72E-40	3.35E-37
OAS2	2.127227	8.866427	22.48349	4.29E-40	3.47E-37
MT2A	1.951413	10.89418	22.24169	1.03E-39	7.57E-37
SIGLEC1	2.302852	7.867264	22.18394	1.27E-39	8.56E-37
UBE2L6	1.559442	10.02154	21.69653	7.58E-39	4.71E-36
MX2	2.011969	9.571431	20.83935	1.87E-37	1.08E-34
IFI44L	3.284203	9.792911	20.81931	2.02E-37	1.09E-34
HERC5	2.897219	8.481786	19.95158	5.67E-36	2.86E-33
IFIH1	2.507172	6.663009	19.84564	8.57E-36	3.85E-33
IFIT3	3.317893	9.570969	19.84532	8.58E-36	3.85E-33
OAS1	2.478011	8.609798	19.79846	1.03E-35	4.34E-33
OAS3	2.483065	9.430131	19.78798	1.07E-35	4.34E-33
RPL15	-1.16067	11.20305	-19.5124	3.17E-35	1.22E-32
OASL	2.412241	8.245835	19.4683	3.78E-35	1.39E-32
PLSCR1	2.201189	10.15494	19.35737	5.86E-35	2.06E-32
IFI44	2.405553	9.473641	19.19805	1.10E-34	3.72E-32
IFITM2	1.505494	12.40325	19.17699	1.20E-34	3.88E-32
IFIT1	3.999163	9.837913	19.1311	1.44E-34	4.48E-32
CXCL10	4.0529	9.119427	18.88586	3.86E-34	1.15E-31
GBP1	2.380375	9.186476	18.70799	7.90E-34	2.28E-31
LY6E	2.02709	9.159572	18.66086	9.57E-34	2.67E-31
MT1H	1.26982	9.661672	18.5714	1.38E-33	3.71E-31
CD1C	-1.23557	7.269684	-18.2235	5.70E-33	1.44E-30
ISG20	1.52729	9.705354	18.22321	5.71E-33	1.44E-30
APOL6	1.189236	7.981179	17.58166	8.17E-32	2.00E-29
AIM2	2.067903	7.722554	17.52081	1.05E-31	2.43E-29
CCL2	3.346448	7.172573	17.24494	3.37E-31	7.57E-29
LAP3	1.750951	9.793299	17.07124	7.04E-31	1.54E-28
TNFSF10	2.350192	10.43542	16.87472	1.63E-30	3.46E-28
RTP4	1.924457	7.737149	16.74624	2.82E-30	5.85E-28
PSMB9	1.051836	10.27339	16.57812	5.82E-30	1.15E-27
ADAR	1.079869	10.49261	16.39155	1.31E-29	2.46E-27
LAMP3	2.075722	5.853532	16.34752	1.58E-29	2.90E-27
CTSL	1.599636	7.970051	16.03245	6.25E-29	1.12E-26
NMI	1.402723	9.572392	15.99273	7.45E-29	1.31E-26
MT1X	1.319141	8.883495	15.96424	8.44E-29	1.45E-26
RNASE2	1.87242	9.568733	15.67609	3.01E-28	4.96E-26
SCO2	1.61904	9.235883	15.57602	4.69E-28	7.58E-26
HESX1	2.008577	5.633309	15.52896	5.78E-28	9.16E-26
GCH1	1.253618	10.14207	15.2474	2.03E-27	3.10E-25
CCR1	1.414576	9.996939	14.90109	9.68E-27	1.42E-24
TRIM21	1.050794	7.708094	14.72283	2.17E-26	3.14E-24
HIST2H2BE	1.75018	7.834621	14.637	3.21E-26	4.40E-24
MTHFD2	1.20453	8.542944	14.59525	3.88E-26	5.23E-24
TCN2	1.232039	6.59848	14.56144	4.53E-26	6.01E-24
DDX58	2.109104	7.830652	14.46323	7.10E-26	9.12E-24
MS4A4A	2.701242	6.834811	14.43032	8.26E-26	1.04E-23
RPL10A	-1.13017	11.51816	-14.2881	1.59E-25	1.97E-23
C1QB	1.669102	6.30562	14.26897	1.73E-25	2.12E-23

NELL2	-1.74325	8.023461	-14.1698	2.74E-25	3.18E-23
VAMP5	1.410563	7.787231	14.07011	4.34E-25	4.87E-23
TLR7	1.672053	6.564752	14.06648	4.41E-25	4.87E-23
CACNA2D:	-1.39573	5.715858	-14.0644	4.45E-25	4.87E-23
HIST1H2BI	1.586012	7.934041	14.01024	5.72E-25	6.09E-23
IFIT5	1.68002	7.296031	13.86955	1.10E-24	1.14E-22
GMPR	1.482376	5.967208	13.84333	1.24E-24	1.27E-22
EIF3F	-1.02561	10.42028	-13.833	1.30E-24	1.32E-22
IGF2BP3	1.092435	4.72826	13.79395	1.56E-24	1.52E-22
SLC31A2	1.208286	8.999759	13.52853	5.40E-24	4.75E-22
CCL8	3.426813	6.32016	13.50215	6.12E-24	5.32E-22
WARS	1.472246	10.41746	13.37754	1.10E-23	9.35E-22
H1F0	1.265898	6.921508	13.27722	1.76E-23	1.45E-21
IFI16	1.194928	9.820166	13.19624	2.58E-23	2.01E-21
DYNLT1	1.113984	10.1067	13.11992	3.70E-23	2.85E-21
HIST1H1C	1.207348	6.925952	13.08625	4.34E-23	3.31E-21
EEF2	-1.18925	10.66412	-13.0412	5.38E-23	3.99E-21
TFEC	1.50313	7.847688	12.9964	6.65E-23	4.89E-21
KLRB1	-1.50119	9.117894	-12.8745	1.19E-22	8.65E-21
TRIM38	1.21715	8.038355	12.84329	1.38E-22	9.83E-21
CASP5	1.049703	5.798581	12.73428	2.32E-22	1.61E-20
IL15	1.076455	7.162237	12.70771	2.63E-22	1.80E-20
MYOF	1.457588	7.973198	12.68617	2.91E-22	1.98E-20
MICB	1.108826	7.739298	12.64725	3.51E-22	2.36E-20
EIF4B	-1.23242	8.811701	-12.4442	9.28E-22	6.05E-20
RPS5	-1.04747	11.79002	-12.4033	1.13E-21	7.30E-20
STAT1	1.276812	8.66925	12.37792	1.28E-21	8.19E-20
EIF2AK2	1.05241	7.465406	12.31492	1.73E-21	1.08E-19
CD38	1.564701	7.946727	12.2691	2.15E-21	1.32E-19
ABCA1	1.430788	6.271855	12.22049	2.72E-21	1.64E-19
B4GALT5	1.052384	7.532669	12.09741	4.93E-21	2.87E-19
DYSF	1.072579	7.820747	11.98135	8.65E-21	4.79E-19
NAP1L1	-1.14976	6.837147	-11.9758	8.88E-21	4.88E-19
HPSE	1.229736	7.071722	11.90344	1.26E-20	6.89E-19
JUP	1.052401	6.253511	11.88691	1.37E-20	7.36E-19
LILRB4	1.127184	6.555443	11.83344	1.77E-20	9.42E-19
LILRB2	1.24623	8.91299	11.82692	1.83E-20	9.66E-19
LMO2	1.078096	9.156565	11.77094	2.40E-20	1.25E-18
LILRA3	1.53112	7.80214	11.74442	2.73E-20	1.41E-18
RPLP0	-1.11372	10.80337	-11.7112	3.21E-20	1.64E-18
RTN1	-1.43975	5.004184	-11.6382	4.58E-20	2.30E-18
FPR2	1.448619	6.945157	11.35939	1.78E-19	8.34E-18
C1QA	1.612135	6.597955	11.0999	6.36E-19	2.92E-17
EXT1	1.036319	6.371398	11.03596	8.71E-19	3.98E-17
KMO	1.046783	6.926891	10.93811	1.41E-18	6.32E-17
4-Sep	1.004263	4.815746	10.9225	1.52E-18	6.75E-17
RBM3	-1.36023	7.782048	-10.8108	2.63E-18	1.14E-16
ENPP2	1.047151	4.275735	10.80368	2.73E-18	1.17E-16
C3AR1	1.501524	9.733587	10.61052	7.07E-18	2.87E-16
XAF1	1.807038	8.716581	10.60067	7.42E-18	2.98E-16
CDKN1A	1.167235	8.944735	10.37825	2.22E-17	8.57E-16
ADM	1.559301	7.588803	10.37805	2.23E-17	8.57E-16
H2AFV	-1.20975	6.829831	-10.2648	3.90E-17	1.47E-15
DEFB1	1.360108	5.98619	10.25416	4.11E-17	1.54E-15
ZNF91	-1.34745	7.619867	-10.2166	4.95E-17	1.82E-15
HSPA6	1.025425	7.365093	9.91126	2.24E-16	7.56E-15
CXCL11	1.58894	4.038712	9.787248	4.15E-16	1.36E-14
NFE2	1.276545	7.336118	9.7498	4.99E-16	1.62E-14

IFI6	1.996473	7.536071	9.636758	8.74E-16	2.77E-14
ACSL1	1.312344	8.748151	9.599287	1.05E-15	3.29E-14
P2RY13	1.147945	8.136913	9.508197	1.65E-15	5.01E-14
GRN	1.04644	9.625999	9.4005	2.82E-15	8.35E-14
HIST1H2BI	1.196992	6.377773	9.255074	5.79E-15	1.64E-13
BST2	1.013575	8.399299	9.215145	7.06E-15	1.99E-13
FFAR2	1.851985	7.917569	9.199241	7.64E-15	2.14E-13
IDO1	1.680744	7.07697	9.12246	1.12E-14	3.04E-13
TNFAIP6	2.147784	6.198443	9.057084	1.54E-14	4.15E-13
GZMK	-1.12937	8.772031	-8.93259	2.86E-14	7.50E-13
NFIL3	1.091538	8.706087	8.64432	1.19E-13	2.84E-12
AQP9	1.19124	8.722	8.415927	3.65E-13	8.17E-12
SH2D1A	-1.05844	7.384044	-8.16228	1.27E-12	2.64E-11
GPR183	-1.08453	7.483866	-7.86433	5.42E-12	1.03E-10
IL1RN	1.534816	9.029574	7.860076	5.53E-12	1.04E-10
BCL11B	-1.23617	7.873893	-7.71461	1.12E-11	1.98E-10
ABLIM1	-1.09787	8.473102	-7.56556	2.30E-11	3.88E-10
HIST1H2A(	1.088521	9.751543	7.437137	4.27E-11	6.81E-10
MS4A1	-1.29013	9.040284	-7.39719	5.17E-11	8.16E-10
CDKN1C	1.111179	8.090916	7.199165	1.33E-10	1.97E-09
HSPB1	1.15912	7.225509	6.99872	3.45E-10	4.76E-09
APOBEC3E	1.902764	5.941999	6.691017	1.47E-09	1.82E-08
ITM2A	-1.10435	7.728025	-6.66191	1.68E-09	2.05E-08
LEF1	-1.06671	8.834887	-6.61633	2.07E-09	2.50E-08
MXD1	1.000549	6.492971	6.481558	3.87E-09	4.46E-08
ATF3	1.085395	8.316003	6.239389	1.17E-08	1.27E-07
FCGR3B	1.1655	10.24337	6.214274	1.32E-08	1.40E-07
IL7R	-1.05698	9.753249	-5.90874	5.20E-08	5.00E-07
RRM2	1.181847	7.429349	5.821728	7.66E-08	7.18E-07
CXCR2	1.196109	7.41316	5.505827	3.05E-07	2.58E-06
KCNJ15	1.074866	6.277059	5.02931	2.28E-06	1.63E-05
CXCL8	-1.20115	9.096006	-3.85354	0.00021	0.000947
