# Supplementary material for: Identification of stage-related and severity-related biomarkers and exploration of immune landscape for Dengue by comprehensive analyses
Source: Virol J. 2022 Aug 2;19:130. doi: 10.1186/s12985-022-01853-8 (PMC9344228; doi:10.1186/s12985-022-01853-8)
Supplement: Supplementary file 8 — Additional file 8. Table S2. Differentially expressed genes (DEGs) in the C vs LA group. (C, Convalescent stage; LA, Late Acute stage). [file 12985_2022_1853_MOESM8_ESM.pdf]

Gene	logFC	AveExpr	t	P.Value	adj.P.Val
IFI27	5.52156	8.644821	21.96298	3.75E-44	3.03E-40
RRM2	3.525531	8.971619	20.00354	2.81E-40	1.14E-36
TYMS	2.948119	8.436925	19.04574	2.62E-38	7.07E-35
CD38	2.650206	8.771497	17.77728	1.27E-35	2.58E-32
CACNA2D:	-1.48212	5.51221	-16.8524	1.32E-33	2.13E-30
GMNN	2.197851	7.386768	16.74491	2.27E-33	2.72E-30
PCNA	1.925673	8.890688	16.43415	1.11E-32	1.12E-29
ZWINT	2.467968	7.103607	16.30172	2.19E-32	1.97E-29
UBE2C	1.514975	6.714159	16.20059	3.68E-32	2.98E-29
FABP5	2.039464	7.7019	16.04204	8.34E-32	6.13E-29
CD1C	-1.10077	7.216866	-15.9309	1.48E-31	9.70E-29
TPX2	1.72376	6.219746	15.92119	1.56E-31	9.70E-29
CDC20	2.082387	6.438311	15.87723	1.96E-31	1.13E-28
NUSAP1	2.237412	7.180653	15.79448	3.01E-31	1.62E-28
HIST1H1C	1.18036	7.04075	15.66208	6.00E-31	3.03E-28
CDKN3	2.23152	6.3273	15.58754	8.85E-31	4.21E-28
PTTG1	1.921112	8.866056	15.57089	9.65E-31	4.34E-28
BIRC5	1.784462	6.573373	15.41872	2.14E-30	9.11E-28
GINS2	1.633628	6.762558	15.32243	3.55E-30	1.43E-27
CKS2	2.068417	7.23411	15.10158	1.13E-29	4.17E-27
BUB1	2.165623	5.569953	14.94736	2.56E-29	9.01E-27
MCM6	1.634582	8.123851	14.91364	3.06E-29	1.03E-26
FEN1	1.430699	7.625347	14.86586	3.95E-29	1.28E-26
CCNB2	1.91268	6.84032	14.73312	7.98E-29	2.48E-26
RTN1	-1.5278	4.794751	-14.6146	1.50E-28	4.49E-26
CDC45	1.108236	5.718048	14.50464	2.70E-28	7.78E-26
CCNA2	1.586122	5.599496	14.38396	5.14E-28	1.40E-25
NCAPG	1.743382	5.712428	14.38169	5.20E-28	1.40E-25
TK1	1.133757	5.979789	14.27695	9.12E-28	2.38E-25
CKS1B	1.10935	7.536526	14.2022	1.36E-27	3.44E-25
TOP2A	1.831794	5.525213	14.08834	2.51E-27	5.97E-25
POLE2	1.127223	6.294895	13.84859	9.14E-27	2.11E-24
CDK1	1.852121	5.843353	13.73669	1.67E-26	3.76E-24
MANF	1.311499	8.971208	13.7114	1.92E-26	4.20E-24
HMMR	1.484165	6.315986	13.46795	7.19E-26	1.49E-23
MCM2	1.349752	7.439785	13.46176	7.44E-26	1.50E-23
CDC6	1.039853	5.07311	13.35536	1.33E-25	2.62E-23
TRIP13	1.142252	6.205543	13.29252	1.87E-25	3.52E-23
SAR1B	1.372206	7.471139	13.24967	2.36E-25	4.34E-23
RFC4	1.409818	7.164493	13.17996	3.45E-25	6.21E-23
MKI67	1.119872	5.982507	13.01862	8.34E-25	1.38E-22
AURKA	1.250872	5.519546	13.01247	8.63E-25	1.40E-22
GZMB	1.393964	10.58891	13.00118	9.18E-25	1.46E-22
NDC80	1.489256	4.982188	12.85498	2.04E-24	3.06E-22
STMN1	1.260918	7.576568	12.83596	2.27E-24	3.34E-22
NELL2	-1.15448	8.189404	-12.7193	4.31E-24	6.22E-22
ISG20	1.081013	9.601929	12.70563	4.64E-24	6.58E-22
AURKB	1.119806	6.092521	12.68282	5.26E-24	7.33E-22
NME1	1.096349	8.231963	12.56492	1.01E-23	1.33E-21
SLAMF7	1.292258	7.238835	12.50405	1.41E-23	1.72E-21
MTHFD2	1.009282	8.555925	12.49436	1.48E-23	1.79E-21
APOBEC3E	1.964862	6.186044	12.30455	4.22E-23	4.95E-21
XBP1	1.473855	8.648047	12.29365	4.48E-23	5.13E-21
RACGAP1	1.473986	5.448512	12.29288	4.50E-23	5.13E-21
MYL6B	1.004823	6.730841	12.24681	5.81E-23	6.43E-21
SRP54	1.045065	7.894293	12.24104	5.99E-23	6.55E-21
PBK	1.823904	5.134479	12.20099	7.48E-23	7.96E-21

SUB1	1.171644	11.17957	12.20083	7.49E-23	7.96E-21
TUBG1	1.019991	5.457503	12.18972	7.96E-23	8.29E-21
BUB1B	1.395615	5.991457	12.18888	8.00E-23	8.29E-21
KIF4A	1.057755	5.482926	12.17669	8.55E-23	8.75E-21
FKBP11	1.678198	8.992823	12.10107	1.30E-22	1.31E-20
CASP3	1.101391	7.199253	12.08905	1.39E-22	1.37E-20
EZH2	1.3796	5.727346	11.95416	2.93E-22	2.75E-20
GGH	2.023446	5.60424	11.9381	3.20E-22	2.97E-20
IL13RA1	-1.02305	6.671867	-11.9127	3.68E-22	3.31E-20
PRC1	1.410613	5.400473	11.81559	6.31E-22	5.48E-20
MAN1A1	1.387368	8.443629	11.77263	8.00E-22	6.74E-20
IDH2	1.092347	8.539302	11.75972	8.59E-22	7.16E-20
CENPE	1.043623	5.481305	11.68973	1.27E-21	1.01E-19
KIF20A	1.025363	4.94023	11.63449	1.72E-21	1.35E-19
CHEK1	1.261228	4.397335	11.59559	2.14E-21	1.64E-19
TNFRSF17	2.690346	7.666701	11.30118	1.09E-20	7.50E-19
CTNNAL1	1.0025	5.749705	11.12473	2.92E-20	1.92E-18
KIF15	1.183446	4.956528	10.9309	8.57E-20	5.46E-18
TTK	1.338076	4.649877	10.91396	9.42E-20	5.90E-18
CPVL	-1.16572	8.937595	-10.8848	1.11E-19	6.79E-18
KIF11	1.789853	4.841394	10.78379	1.94E-19	1.15E-17
PRDX4	1.360686	8.241621	10.75858	2.23E-19	1.30E-17
SMC4	1.170422	7.811149	10.62125	4.79E-19	2.71E-17
UAP1	1.617443	7.692249	10.57313	6.26E-19	3.38E-17
IGJ	1.714038	9.991761	10.36782	1.96E-18	9.60E-17
FBXO5	1.107709	4.516082	10.30362	2.80E-18	1.34E-16
PAICS	1.003506	7.687267	9.995101	1.55E-17	6.53E-16
LAP3	1.077039	9.576823	9.960083	1.88E-17	7.76E-16
MX1	1.365149	10.03266	9.958509	1.90E-17	7.79E-16
CLEC4A	-1.13747	7.023307	-9.95431	1.94E-17	7.93E-16
MAD2L1	1.500736	5.224085	9.850049	3.46E-17	1.33E-15
OAS2	1.00151	8.418226	9.845616	3.55E-17	1.35E-15
MCM5	1.001125	7.813064	9.824755	3.98E-17	1.50E-15
ELL2	1.508726	6.319344	9.786769	4.91E-17	1.84E-15
USP18	1.185739	6.358102	9.710371	7.49E-17	2.67E-15
SLC1A4	1.17233	5.899039	9.704561	7.74E-17	2.74E-15
RRM1	1.132432	8.001128	9.679464	8.89E-17	3.10E-15
HIST1H2BH	1.022769	6.402619	9.471446	2.80E-16	8.78E-15
ITM2C	1.295696	8.151864	9.169632	1.47E-15	3.95E-14
CDKN1C	-1.41183	6.689408	-8.69031	2.01E-14	4.45E-13
SDF2L1	1.207478	7.330406	8.660466	2.37E-14	5.14E-13
DUSP5	1.110586	7.64696	8.204015	2.78E-13	5.06E-12
MS4A1	-1.24015	8.930373	-8.17972	3.16E-13	5.70E-12
PPIB	1.113508	9.993215	8.164111	3.44E-13	6.14E-12
ISG15	1.407873	9.907618	8.150028	3.71E-13	6.53E-12
GBP1	1.304719	8.795961	8.067009	5.78E-13	9.90E-12
IFI44	1.123536	8.961361	7.877251	1.58E-12	2.54E-11
IFI44L	1.529007	9.090553	7.502773	1.13E-11	1.65E-10
CCR2	1.028547	9.244754	7.36177	2.36E-11	3.23E-10
SGK1	-1.00816	9.765077	-6.86206	3.05E-10	3.49E-09
TREM1	-1.05895	8.178988	-6.72387	6.10E-10	6.56E-09
PPP1R15A	-1.01378	8.47655	-6.33852	4.10E-09	3.85E-08
TPD52	1.006421	7.989509	6.275403	5.57E-09	5.05E-08
SERPING1	1.378765	6.404018	6.070444	1.49E-08	1.24E-07
HBB	-1.72678	11.49115	-5.72609	7.56E-08	5.47E-07
IFI6	1.005302	7.154847	5.609144	1.30E-07	9.06E-07
CD83	-1.18384	8.294901	-5.52731	1.89E-07	1.28E-06
IER3	-1.29213	8.828202	-5.47374	2.41E-07	1.61E-06

PTGS2	-1.46945	7.463654	-5.45668	2.60E-07	1.73E-06
LGALS2	-1.28155	7.802286	-5.41866	3.08E-07	2.03E-06
IFIT1	1.281082	8.632264	5.349381	4.21E-07	2.69E-06
CXCL8	-1.66039	8.688539	-5.0144	1.83E-06	1.04E-05
IL1B	-1.45551	9.167138	-4.38266	2.50E-05	0.000114
G0S2	-1.27993	8.223953	-4.14258	6.37E-05	0.000265
TNF	-1.07436	7.296669	-4.03453	9.59E-05	0.000387
CXCL10	1.007338	7.72204	3.471584	0.000717	0.002353
CXCL2	-1.06024	6.969738	-3.17123	0.001921	0.005624
