# Supplementary material for: Identification of stage-related and severity-related biomarkers and exploration of immune landscape for Dengue by comprehensive analyses
Source: Virol J. 2022 Aug 2;19:130. doi: 10.1186/s12985-022-01853-8 (PMC9344228; doi:10.1186/s12985-022-01853-8)
Supplement: Supplementary file 9 — Additional file 9. Table S3. Differentially expressed genes (DEGs) in the EA vs LA group. (EA, Early Acute stage; LA, Late Acute stage). [file 12985_2022_1853_MOESM9_ESM.pdf]

Gene	logFC	AveExpr	t	P.Value	adj.P.Val
CDKN1C	-2.52301	7.117522	-14.0209	4.32E-27	3.49E-23
NME1	1.308282	8.154468	13.40445	1.19E-25	4.83E-22
RPLP0	1.26228	11.00854	13.03094	9.08E-25	2.45E-21
MX2	-1.78483	9.502232	-12.899	1.86E-24	3.77E-21
HERC5	-2.11202	8.660831	-12.7626	3.92E-24	6.34E-21
CCL8	-3.09414	6.169335	-12.6116	8.95E-24	1.03E-20
HESX1	-1.60032	5.674644	-12.4619	2.03E-23	1.83E-20
CTSL	-1.45738	7.891716	-12.3799	3.19E-23	2.34E-20
OASL	-1.6468	8.462847	-12.3424	3.92E-23	2.64E-20
JUP	-1.11981	6.104034	-12.2225	7.57E-23	4.71E-20
MS4A4A	-2.67346	6.573296	-12.1836	9.37E-23	5.36E-20
LDHB	1.011155	11.00549	12.1728	9.95E-23	5.36E-20
TNFSF10	-1.90895	10.46161	-12.1035	1.46E-22	6.92E-20
BIRC5	1.469231	6.70581	12.01187	2.41E-22	1.08E-19
CDKN3	1.876037	6.477749	11.92407	3.91E-22	1.58E-19
TPX2	1.353961	6.37325	11.77257	9.02E-22	2.80E-19
TYMS	1.996401	8.824503	11.64386	1.83E-21	5.49E-19
PTTG1	1.576912	9.010526	11.63151	1.96E-21	5.67E-19
FABP5	1.669117	7.857207	11.5958	2.39E-21	6.06E-19
IFIT1	-2.71808	10.20504	-11.5954	2.40E-21	6.06E-19
GINS2	1.301327	6.900922	11.57278	2.72E-21	6.64E-19
LILRA3	-1.71561	7.532098	-11.5541	3.01E-21	6.96E-19
ZWINT	1.921421	7.330079	11.54432	3.18E-21	7.14E-19
C3AR1	-1.64657	9.49059	-11.5386	3.28E-21	7.17E-19
STMN1	1.2057	7.604534	11.50537	3.94E-21	8.39E-19
CDC20	1.66083	6.61389	11.44921	5.38E-21	1.04E-18
LILRB2	-1.61848	8.558089	-11.3951	7.25E-21	1.27E-18
RRM2	2.343684	9.452233	11.37726	8.01E-21	1.35E-18
SIGLEC1	-1.52735	8.101672	-11.2719	1.43E-20	2.32E-18
PAICS	1.301826	7.575471	11.23522	1.76E-20	2.58E-18
MX1	-1.49354	11.15918	-11.2047	2.08E-20	3.00E-18
CDKN1A	-1.17565	8.819312	-11.1962	2.18E-20	3.09E-18
ISG15	-1.94774	11.22898	-11.1858	2.31E-20	3.22E-18
CCR1	-1.29103	9.926298	-11.1563	2.72E-20	3.68E-18
CCNB2	1.55254	6.990991	11.14623	2.87E-20	3.81E-18
OAS1	-1.48414	8.958996	-11.1297	3.15E-20	4.04E-18
CASP5	-1.03988	5.696363	-11.1219	3.29E-20	4.15E-18
OAS3	-1.8135	9.581522	-11.0794	4.16E-20	4.94E-18
OAS2	-1.12572	9.256425	-11.0612	4.60E-20	5.39E-18
IFIH1	-1.66745	6.915425	-11.0367	5.27E-20	5.92E-18
GLUL	-1.18828	9.830661	-10.9783	7.28E-20	7.64E-18
LMO2	-1.3973	8.851264	-10.9631	7.92E-20	8.12E-18
HPSE	-1.16033	6.987195	-10.9569	8.19E-20	8.18E-18
IFIT3	-2.47056	9.744453	-10.9268	9.68E-20	9.55E-18
PLSCR1	-1.37881	10.42835	-10.921	9.99E-20	9.74E-18
FEN1	1.152198	7.741619	10.83042	1.65E-19	1.52E-17
CCL2	-2.70575	7.217413	-10.782	2.16E-19	1.90E-17
SLC31A2	-1.35815	8.784053	-10.7787	2.20E-19	1.90E-17
TOP2A	1.496078	5.665911	10.76588	2.36E-19	2.01E-17
GMPR	-1.10672	6.042945	-10.7563	2.49E-19	2.07E-17
TLR7	-1.33849	6.595328	-10.7091	3.23E-19	2.54E-17
LAMP3	-1.63293	5.908953	-10.709	3.23E-19	2.54E-17
DDX58	-1.63011	7.904656	-10.6913	3.56E-19	2.77E-17
CCNA2	1.241109	5.7426	10.62801	5.06E-19	3.75E-17
RFC4	1.260158	7.230198	10.61481	5.45E-19	3.94E-17
SLC7A7	-1.03029	9.709052	-10.6144	5.46E-19	3.94E-17
UBE2C	1.047053	6.905045	10.58874	6.29E-19	4.42E-17

H1F0	-1.09817	6.893067	-10.5237	9.02E-19	6.01E-17
CD27	1.258945	9.05427	10.51714	9.35E-19	6.10E-17
IL15	-1.09503	7.03999	-10.4885	1.10E-18	6.98E-17
IFITM3	-1.26108	12.29495	-10.4767	1.17E-18	7.39E-17
IRF7	-1.5741	9.483542	-10.4534	1.33E-18	8.34E-17
MARCKS	-1.60563	9.139907	-10.4478	1.37E-18	8.54E-17
NUSAP1	1.600358	7.441414	10.43507	1.47E-18	8.95E-17
NCAPG	1.391867	5.858869	10.41053	1.69E-18	1.02E-16
CDK1	1.490502	5.994299	10.38552	1.94E-18	1.15E-16
GMNN	1.508936	7.667643	10.37969	2.00E-18	1.18E-16
IFI44	-1.28202	9.909184	-10.362	2.21E-18	1.28E-16
ABCA1	-1.29116	6.209325	-10.358	2.26E-18	1.29E-16
BUB1	1.722858	5.754257	10.34474	2.43E-18	1.38E-16
IFIT5	-1.27597	7.368671	-10.3317	2.61E-18	1.48E-16
MT2A	-1.0999	11.21105	-10.2467	4.17E-18	2.24E-16
TLR1	-1.30133	7.402487	-10.2438	4.24E-18	2.26E-16
HMMR	1.20922	6.431134	10.21518	4.97E-18	2.58E-16
BAG2	1.064192	5.40003	10.21046	5.10E-18	2.63E-16
SERPING1	-2.3875	7.88607	-10.1737	6.25E-18	3.12E-16
CFD	-1.33459	8.646049	-10.1541	6.96E-18	3.41E-16
PRDX2	1.322803	8.268121	10.13711	7.65E-18	3.68E-16
BUB1B	1.210771	6.070871	10.13087	7.92E-18	3.79E-16
FGL2	-1.36032	10.07694	-10.09	9.92E-18	4.66E-16
SERPINA1	-1.50694	10.58732	-10.0878	1.00E-17	4.69E-16
SMC4	1.142665	7.827904	10.08051	1.05E-17	4.86E-16
IFI44L	-1.7552	10.38455	-10.0748	1.08E-17	4.99E-16
MCM2	1.116184	7.538052	10.06134	1.16E-17	5.25E-16
UBE2L6	-1.0225	10.18745	-10.0282	1.40E-17	6.13E-16
DEFB1	-1.22434	5.928594	-10.0254	1.42E-17	6.20E-16
KMO	-1.12955	6.76865	-10.0077	1.56E-17	6.76E-16
EMR2	-1.07307	5.886826	-9.99418	1.68E-17	7.21E-16
SCO2	-1.21407	9.315363	-9.95529	2.09E-17	8.79E-16
USP18	-1.37717	7.367871	-9.93143	2.38E-17	9.82E-16
RRM1	1.227741	7.969492	9.908762	2.70E-17	1.10E-15
TFEC	-1.58305	7.644142	-9.90574	2.74E-17	1.12E-15
MCM6	1.197181	8.303384	9.841683	3.91E-17	1.54E-15
VRK1	1.135989	7.051338	9.823871	4.31E-17	1.67E-15
PILRA	-1.52534	6.628331	-9.75387	6.34E-17	2.28E-15
LYN	-1.06928	8.442868	-9.75106	6.44E-17	2.30E-15
LILRA2	-1.23832	8.099664	-9.73875	6.89E-17	2.44E-15
PCNA	1.176657	9.193734	9.738251	6.91E-17	2.44E-15
PBK	1.549391	5.251167	9.728631	7.28E-17	2.54E-15
NAGK	-1.03761	9.794701	-9.70866	8.13E-17	2.81E-15
CCNC	1.014637	9.008343	9.680733	9.48E-17	3.22E-15
IFITM2	-1.04877	12.52591	-9.61874	1.33E-16	4.46E-15
MCM5	1.043514	7.801495	9.611615	1.39E-16	4.62E-15
CD1D	-1.07079	7.435717	-9.60229	1.46E-16	4.76E-15
C1QBP	1.051366	7.664966	9.58929	1.57E-16	5.01E-15
HMGB1	1.205197	9.283921	9.577935	1.67E-16	5.27E-15
GZMK	1.362412	9.030202	9.574451	1.70E-16	5.35E-15
LGALS3BP	-1.44236	8.119282	-9.54532	2.00E-16	6.23E-15
PRC1	1.177253	5.498965	9.504794	2.50E-16	7.58E-15
DYNLT1	-1.02805	10.04416	-9.43961	3.57E-16	1.05E-14
CCT2	1.05606	7.943694	9.438279	3.60E-16	1.05E-14
CKS2	1.304068	7.543879	9.405244	4.31E-16	1.21E-14
NOD2	-1.0131	6.411822	-9.3676	5.30E-16	1.47E-14
GCH1	-1.00726	10.16273	-9.36269	5.44E-16	1.51E-14
LPAR6	-1.09735	8.559657	-9.34993	5.84E-16	1.59E-14

KIF11	1.617239	4.917999	9.349728	5.84E-16	1.59E-14
CXCL10	-3.04556	9.314491	-9.3396	6.18E-16	1.66E-14
LY6E	-1.1484	9.485178	-9.28605	8.28E-16	2.17E-14
FKBP11	1.451111	9.090203	9.237625	1.08E-15	2.77E-14
GGH	1.695673	5.74279	9.175828	1.51E-15	3.75E-14
TYROBP	-1.17127	11.21721	-9.15563	1.69E-15	4.13E-14
EZH2	1.112507	5.838893	9.144549	1.80E-15	4.35E-14
KIF15	1.020517	5.026292	9.133216	1.91E-15	4.57E-14
TLR2	-1.09246	9.181738	-9.1267	1.98E-15	4.71E-14
FCN1	-1.24752	11.12488	-9.12395	2.01E-15	4.75E-14
FCER1G	-1.11522	10.68591	-9.09198	2.39E-15	5.56E-14
P2RY13	-1.44395	7.838526	-9.08721	2.46E-15	5.64E-14
CHEK1	1.046983	4.487588	9.082052	2.53E-15	5.77E-14
ASNS	1.046664	6.537342	9.054678	2.93E-15	6.59E-14
ITGB3BP	1.060638	7.064659	9.031115	3.34E-15	7.41E-14
SLC11A1	-1.00772	7.776163	-9.0085	3.77E-15	8.24E-14
IL1RN	-1.87304	8.665627	-9.00498	3.85E-15	8.34E-14
CD14	-1.1334	10.68261	-8.93102	5.75E-15	1.21E-13
CEBPB	-1.10093	10.79588	-8.87754	7.69E-15	1.58E-13
TNFRSF17	2.426982	7.783378	8.86524	8.23E-15	1.68E-13
GZMA	1.012663	10.35691	8.86249	8.35E-15	1.69E-13
RACGAP1	1.092971	5.605154	8.786103	1.26E-14	2.45E-13
MNDA	-1.07125	10.86024	-8.75839	1.47E-14	2.79E-13
FPR1	-1.13368	10.22488	-8.7226	1.78E-14	3.30E-13
SH2D1A	1.147508	7.547321	8.716353	1.84E-14	3.41E-13
MAD2L1	1.412745	5.266094	8.69041	2.12E-14	3.87E-13
TTK	1.110098	4.745895	8.621628	3.08E-14	5.45E-13
NDC80	1.049353	5.161971	8.62072	3.09E-14	5.46E-13
AIM2	-1.11333	8.090113	-8.61541	3.19E-14	5.55E-13
TPD52	1.488545	7.805737	8.496365	6.05E-14	1.01E-12
MAN1A1	1.074948	8.572969	8.493202	6.16E-14	1.02E-12
HCK	-1.06518	9.337898	-8.48466	6.45E-14	1.06E-12
FFAR2	-1.80117	7.757599	-8.4457	7.95E-14	1.28E-12
ITM2C	1.295349	8.158515	8.412762	9.49E-14	1.49E-12
MS4A6A	-1.0682	9.728285	-8.37244	1.18E-13	1.83E-12
MAFB	-1.23699	9.951336	-8.36595	1.22E-13	1.88E-12
UAP1	1.505741	7.74413	8.312678	1.62E-13	2.46E-12
XBP1	1.156948	8.779579	8.228221	2.55E-13	3.77E-12
ADM	-1.29468	7.589066	-8.22817	2.55E-13	3.77E-12
FCGR2A	-1.05673	7.782691	-8.18329	3.24E-13	4.68E-12
CST3	-1.18744	9.509897	-8.16812	3.52E-13	5.03E-12
SDF2L1	1.200359	7.339265	8.069755	5.94E-13	8.01E-12
AQP9	-1.40749	8.467666	-8.06411	6.12E-13	8.22E-12
C5AR1	-1.14687	8.702863	-8.03823	7.02E-13	9.29E-12
GRN	-1.05857	9.510762	-7.99127	9.01E-13	1.17E-11
DUSP6	-1.08295	9.525288	-7.98864	9.14E-13	1.18E-11
FPR2	-1.21008	6.940957	-7.94829	1.13E-12	1.42E-11
PPIB	1.159518	9.980792	7.817819	2.25E-12	2.66E-11
NFIL3	-1.13323	8.568218	-7.80068	2.47E-12	2.90E-11
RBM3	1.12246	7.777606	7.676609	4.74E-12	5.27E-11
HSPA13	1.122917	6.444764	7.659735	5.17E-12	5.71E-11
CD8A	1.018953	9.278135	7.643814	5.62E-12	6.14E-11
HIST2H2BE	-1.19009	7.994953	-7.63808	5.79E-12	6.30E-11
IGJ	1.370818	10.13481	7.588613	7.50E-12	7.97E-11
NFE2	-1.14875	7.282287	-7.42249	1.78E-11	1.78E-10
BCL2A1	-1.33421	9.534002	-7.34175	2.70E-11	2.63E-10
KLF4	-1.02042	8.916127	-7.28815	3.57E-11	3.38E-10
MYOF	-1.23465	7.958585	-7.26789	3.96E-11	3.72E-10

RNASE2	-1.01233	9.898965	-7.12856	8.09E-11	7.15E-10
MAP3K7CL	-1.45621	7.404855	-7.1014	9.29E-11	8.12E-10
ACSL1	-1.22671	8.664983	-6.87305	2.96E-10	2.38E-09
CD38	1.085505	9.397662	6.830154	3.67E-10	2.89E-09
ELL2	1.159401	6.463749	6.79539	4.37E-10	3.41E-09
XAF1	-1.10039	8.960207	-6.71803	6.43E-10	4.85E-09
GBP1	-1.07566	9.734835	-6.67365	8.03E-10	5.96E-09
ATF3	-1.30137	8.072747	-6.59948	1.16E-09	8.38E-09
HSPB1	-1.2385	7.05775	-6.42443	2.75E-09	1.85E-08
CXCL11	-1.1645	4.133141	-6.2098	7.80E-09	4.94E-08
CLU	-1.03889	7.862656	-6.08215	1.44E-08	8.87E-08
SPI1	-1.07675	6.892757	-6.00892	2.04E-08	1.23E-07
FCGR3B	-1.2471	10.0736	-5.96325	2.53E-08	1.50E-07
NAMPT	-1.07217	8.039961	-5.92326	3.05E-08	1.79E-07
TNFAIP6	-1.56729	6.330206	-5.88337	3.69E-08	2.15E-07
HCAR3	-1.31367	8.300846	-5.87539	3.83E-08	2.22E-07
CXCR2	-1.228	7.270478	-5.56584	1.60E-07	8.45E-07
HBB	-1.57827	11.42431	-5.01836	1.82E-06	8.42E-06
IDO1	-1.14461	7.229886	-4.89475	3.08E-06	1.39E-05
PF4	-1.0234	10.09788	-4.77599	5.08E-06	2.21E-05
PPBP	-1.00002	10.08721	-4.40564	2.30E-05	9.00E-05
MAFF	-1.07547	6.67012	-4.36346	2.72E-05	0.000105
