# Supplementary material for: Identification of stage-related and severity-related biomarkers and exploration of immune landscape for Dengue by comprehensive analyses
Source: Virol J. 2022 Aug 2;19:130. doi: 10.1186/s12985-022-01853-8 (PMC9344228; doi:10.1186/s12985-022-01853-8)
Supplement: Supplementary file 10 — Additional file 10. Table S4. Differentially expressed genes (DEGs) from comparing Dengue Hemorrhagic Fever (DHF) with Dengue Fever (DF) in EA group. (EA, Early Acute stage). [file 12985_2022_1853_MOESM10_ESM.pdf]

Gene	logFC	AveExpr	t	P.Value	adj.P.Val
PSPH	-1.05274	5.350858	-4.28553	0.000209	0.678119
CACNA2D:	-1.314	6.493936	-4.19824	0.000264	0.678119
LOC28608	-1.79684	4.594629	-4.10968	0.000333	0.678119
POMZP3	-1.12563	6.575035	-3.99139	0.000456	0.678119
PLA2G7	-1.09606	6.933809	-3.94701	0.000512	0.678119
IFNB1	1.280757	4.985103	3.907076	0.000569	0.678119
HLA-DQB1	-1.70985	7.250014	-3.81223	0.00073	0.678119
FRG1B	1.636674	6.353221	3.743664	0.000873	0.678119
CPA3	-1.02361	6.811063	-3.42251	0.002001	0.678119
MYOM2	-2.11089	8.487519	-3.41684	0.00203	0.678119
ZNF595	1.195492	5.346245	3.315802	0.002625	0.678119
DNAAF2	-1.15157	7.518239	-3.23192	0.003242	0.678119
SCOC	-1.06214	8.413396	-3.16245	0.003858	0.678119
MEFV	1.1288	7.472897	3.158333	0.003897	0.678119
LOC10027	1.019662	4.258133	3.070053	0.004851	0.678119
HEPACAM	1.223259	4.351208	2.971608	0.006177	0.678119
TMED2	-1.22813	8.98699	-2.90421	0.007276	0.678119
ZNF600	-1.06721	8.232449	-2.84505	0.008392	0.678119
AC092192.	1.072606	4.670935	2.737589	0.010842	0.678119
CTD-2076I	1.045998	4.12209	2.590673	0.015289	0.678119
CEACAM6	1.046368	6.474206	2.484438	0.019505	0.678119
RP1-142L7	1.152275	4.440718	2.460907	0.020574	0.678119
LOC10050	1.051809	4.003469	2.448371	0.021165	0.678119
ZNF528	1.162024	5.566906	2.43086	0.022017	0.678119
LOC10192	1.039873	5.195303	2.311623	0.028705	0.681467
APOBEC3E	-1.74979	8.936359	-2.28829	0.030213	0.681467
ALDH1A1	-1.06225	8.782954	-2.20854	0.035924	0.681467
DEFA4	1.128427	7.839452	2.201699	0.036456	0.681467
TCN1	1.00889	8.040293	2.08227	0.046969	0.688815
TPTE2P6	1.05676	4.036277	2.055161	0.049702	0.688815
