# Supplementary material for: Identification of stage-related and severity-related biomarkers and exploration of immune landscape for Dengue by comprehensive analyses
Source: Virol J. 2022 Aug 2;19:130. doi: 10.1186/s12985-022-01853-8 (PMC9344228; doi:10.1186/s12985-022-01853-8)
Supplement: Supplementary file 11 — Additional file 11. Table S5. Differentially expressed genes (DEGs) from comparing Dengue Hemorrhagic Fever (DHF) with Dengue Fever (DF) in LA group. (LA, Late Acute stage). [file 12985_2022_1853_MOESM11_ESM.pdf]

Gene	logFC	AveExpr	t	P.Value	adj.P.Val
ZNF595	1.193134	5.36667	5.022812	6.32E-06	0.002443
RTN1	-1.3367	6.45682	-4.97101	7.57E-06	0.00245
HP	1.578049	8.084475	4.862371	1.11E-05	0.002567
MIR3916	-1.01701	6.340038	-4.83356	1.22E-05	0.002673
MS4A4A	1.241787	8.024534	4.642263	2.36E-05	0.003705
S100P	1.457164	9.413366	4.574467	2.98E-05	0.004159
OPHN1	1.564741	10.67469	4.451056	4.52E-05	0.005154
FRG1B	1.399552	6.116104	4.390788	5.54E-05	0.005794
LOC28608	-1.05459	3.825435	-4.37992	5.74E-05	0.005923
CLIC3	-1.02728	8.079148	-4.21555	9.92E-05	0.00754
ADM	1.228782	9.292053	4.104148	0.000143	0.008876
BTK	1.140897	8.71469	4.067115	0.000161	0.009491
LOC10192	1.063818	5.234987	3.951607	0.000234	0.011449
IFIT1	1.310688	11.16301	3.693612	0.00053	0.017157
SLPI	1.031344	6.913368	3.652394	0.000603	0.018355
MMP8	1.747531	6.876295	3.606651	0.000694	0.0198
IFI27	1.447463	12.73768	3.598701	0.000711	0.020131
CAMP	1.353278	9.537819	3.5624	0.000795	0.021303
ERP29	1.094691	11.43245	3.498645	0.000965	0.023539
MCEMP1	1.019063	9.679475	3.405358	0.001278	0.026602
HERC5	1.000955	9.887843	3.282204	0.001841	0.032626
FOLR3	1.229563	7.244607	3.264818	0.001937	0.03356
IER3	1.384838	10.20483	3.261772	0.001955	0.033615
IL6	1.324535	7.487933	3.261639	0.001955	0.033615
TNF	1.514766	8.620199	3.219011	0.002214	0.036266
RETN	1.103893	8.056399	3.199903	0.00234	0.037274
LCN2	1.359391	8.669229	3.099847	0.003118	0.044048
S100A12	1.491821	11.69217	3.084341	0.003259	0.045005
RNASE3	1.073434	7.91796	3.072548	0.00337	0.046006
IFI44L	1.139602	11.0324	3.072059	0.003374	0.046041
ELANE	1.232331	6.238481	3.043503	0.003658	0.048569
USP9Y	-1.57888	5.023027	-3.0116	0.004	0.051254
OLFM4	1.433727	6.08808	2.915986	0.005216	0.05924
BCL2A1	1.218555	11.0354	2.902498	0.005412	0.060471
RNASE2	1.055133	11.39996	2.872292	0.005878	0.063235
CCL8	1.308832	6.358116	2.815611	0.006855	0.06949
TXLNGY	-1.51002	5.390662	-2.81022	0.006955	0.069917
ANXA3	1.122934	7.500534	2.746055	0.008257	0.077974
TNFAIP6	1.228966	8.102012	2.741071	0.008367	0.078582
KDM5D	-1.73947	5.936435	-2.69024	0.009568	0.085545
TCN1	1.051903	7.595229	2.645378	0.010758	0.091574
RSAD2	1.041707	9.469706	2.635466	0.011038	0.093029
CMPK2	1.032492	10.58084	2.620907	0.011462	0.094951
SERPINB2	1.178727	7.332759	2.591172	0.012375	0.099614
G0S2	1.068148	9.782677	2.564503	0.013249	0.10425
XIST	1.606529	7.072945	2.563435	0.013285	0.104307
EIF1AY	-2.11029	5.906054	-2.56265	0.013312	0.104441
CCL2	1.283445	6.96092	2.561802	0.013341	0.104515
CEACAM8	1.381081	7.472643	2.521548	0.014776	0.111329
CXCL3	1.346672	7.121005	2.519334	0.014859	0.111642
CXCL8	1.314063	10.97158	2.515313	0.01501	0.112504
DDX3Y	-1.14022	5.055542	-2.50074	0.015572	0.114806
LTF	1.258821	9.415118	2.462011	0.017156	0.121644
IFI6	1.131356	11.97862	2.418494	0.019109	0.129352
ANKRD22	1.081901	8.433552	2.386745	0.020658	0.135309
IL1B	1.292196	10.41642	2.375311	0.021243	0.13713
DEFA4	1.099808	8.147578	2.304297	0.025219	0.151956

RPS4Y1	-2.20362	8.337062	-2.21703	0.031002	0.172046
